# A prediction model for platinum-resistant recurrence of ovarian cancer was established using multimodal artificial intelligence machine learning methods

**DOI:** 10.3389/fmed.2026.1842344

**Published:** 2026-07-08

**Authors:** Xuanxuan Zhao, Ling Ma, Peiquan Li, Zhijun Hu, Yue Ding, Qing Liu, Kaijiang Liu

**Affiliations:** 1 Department of Gynaecological Oncology, Ren Ji Hospital, School of Medicine, Shanghai Jiao Tong University, Shanghai, China; 2 Shanghai Jiao Tong University, Shanghai, China

**Keywords:** artificial intelligence, machine learning, multimodal prediction model, ovarian cancer, platinum-resistant recurrence, recurrence prediction

## Abstract

**Aim:**

To develop and validate a multimodal artificial intelligence (AI)-based prediction model for platinum-resistant recurrence in ovarian cancer by integrating clinical data, medical imaging, and medical knowledge resources, with the goal of improving early risk stratification and supporting individualized treatment decisions. This exploratory proof-of-concept study aims to assess the feasibility of multimodal fusion for this task; no external validation has been performed.

**Methods:**

This study collected multimodal data from ovarian cancer patients, including clinical records from 214 patients treated at Renji Hospital affiliated to Shanghai Jiao Tong University School of Medicine between June 2020 and January 2025, imaging data from 218 patients comprising 5,053 CT and MRI images, and 1,000 high-quality medical literature sources published between 2023 and 2025. Patients were classified into a platinum-resistant recurrence group (PROC, *n* = 87, 40.7%) and a non-platinum-resistant recurrence group (NPROC, *n* = 127, 59.3%) according to whether recurrence occurred within 6 months after the last platinum-based chemotherapy. The platinum-free interval (PFI) was used only for outcome definition, not as a predictor. A multimodal prediction framework based on a Mixture of Experts (MoE) architecture was constructed, incorporating a clinical expert model, an imaging expert model, and a medical knowledge expert model. Model performance was evaluated using accuracy, recall, F1 score, and the area under the receiver operating characteristic curve (AUC) with 95% confidence intervals (bootstrap with 1,000 iterations, stratified by cross-validation folds), calibration (Brier score, Hosmer-Lemeshow test, calibration curve), and decision curve analysis (DCA).

**Results:**

The proposed multimodal model demonstrated excellent predictive performance for platinum-resistant recurrence, achieving an accuracy of 0.98 (95% CI: 0.96–1.00), a recall of 0.95 (95% CI: 0.92–0.98), an F1 score of 0.98 (95% CI: 0.97–0.99), and an AUC of 0.96 (95% CI: 0.95–0.97). The model showed good calibration with a Brier score of 0.042 (95% CI: 0.031–0.058) and a Hosmer-Lemeshow test *p*-value of 0.31 (χ^2^ = 11.8, df = 10), indicating no statistically significant lack of fit. These results were superior to those of single-expert and conventional benchmark models. In the clinical expert evaluation, the model achieved an accuracy of 0.83, a recall of 0.81, an F1 score of 0.82, and an AUC of 0.83, showing competitive performance compared with random forest, support vector machine, gradient boosting machine, and Transformer-based models.

**Conclusion:**

This exploratory study demonstrates that multimodal AI integrating clinical, imaging, and knowledge graph data can achieve strong internal predictive performance for platinum-resistant recurrence of ovarian cancer in a single-center retrospective cohort. However, the model is preliminary, has not been externally validated, and is not ready for clinical use. Independent multicenter validation is required before any clinical translation can be considered. This article should be viewed as a hypothesis-generating tool and a methodological proof-of-concept only.

## 1 Introduction

Ovarian cancer is one of the most lethal cancers in the female reproductive system. Its high incidence and mortality rates make it a global public health challenge (1). According to the statistics of the World Health Organization (WHO), the annual incidence rate of ovarian cancer is approximately 5.3 per 100,000, while the mortality rate is as high as 4.0 per 100,000, and this figure increases significantly with age (2). Although the treatment of ovarian cancer has made certain progress through surgical and chemotherapy methods over the past few decades, due to the lack of obvious early symptoms, most patients are diagnosed at an advanced stage, thus missing the best treatment opportunity (3). Currently, the treatment of ovarian cancer mainly relies on platinum-based chemotherapy drugs such as cisplatin and carboplatin, which have achieved certain success in the initial treatment. However, about 70% of patients will experience recurrence after treatment, with the majority being platinum-resistant recurrence (4).

Platinum-resistant recurrence is a major challenge in the treatment of ovarian cancer. Platinum-based drugs have shown significant efficacy in the treatment of ovarian cancer, but as treatment progresses, tumor cells gradually develop resistance to these drugs, leading to a distinct reduction in the efficacy of subsequent treatments (5). The occurrence of platinum-resistant recurrence not only significantly shortens the survival period of patients but also makes subsequent treatments more difficult (6). Currently, there is a lack of an efficient method to predict which patients may experience platinum-resistant recurrence after treatment. Therefore, early identification of the risk of platinum-resistant recurrence and adjustment of treatment strategies based on this is crucial for improving patient survival rates.

Existing methods for predicting platinum-resistant recurrence mainly rely on clinical features, biomarker detection, and imaging assessment (7). However, these methods often have certain limitations and cannot fully exploit the multi-dimensional data of patients. The accuracy of prediction is often affected by data quality and individual differences. In clinical practice, traditional single data sources (such as genomic data, clinical records, and imaging data) often fail to provide comprehensive disease information, affecting the reliability and practicality of prediction results (8). Moreover, existing prediction models lack effective integration methods for multi-source heterogeneous data, limiting their application in clinical settings. Therefore, how to construct a multi-dimensional and multi-data source integrated prediction model to accurately identify patients at high risk of platinum-resistant recurrence is an important topic in current ovarian cancer research.

In recent years, artificial intelligence (AI) technology has been widely applied in the medical field, particularly in cancer diagnosis, prediction, and treatment (9). The emergence of AI has provided a new perspective for medical analysis, especially when dealing with complex multimodal data, demonstrating its great potential. Ovarian cancer, as a highly lethal malignant tumor, has long been a challenge in medical research and clinical treatment. With the continuous development of AI technology, early diagnosis, recurrence prediction, and treatment plan optimization of ovarian cancer have gradually benefited from AI technology, especially machine learning and deep learning methods (10). Currently, AI application in ovarian cancer research mainly focuses on disease diagnosis, personalized treatment plan recommendations, risk prediction, and medical image analysis. For example, convolutional neural networks (CNN) are widely used in the automated diagnosis of ovarian cancer images, which can accurately extract tumor features from CT and MRI data, assisting doctors in more accurate diagnosis and prognosis assessment (11). In genomic data analysis, AI can analyze potential patterns in large-scale genomic data through machine learning algorithms, thereby identifying genetic markers related to the occurrence, development, and recurrence of ovarian cancer (12).

However, the existing AI models for ovarian cancer mostly focus on the analysis of a single data source, such as relying solely on imaging data, clinical records, or a certain part of genomic data for analysis. The limitations of these models lie in their inability to comprehensively consider the multi-dimensional clinical information of patients, resulting in limited application effects in multimodal data fusion and complex clinical decision-making (13). For instance, the existing AI models typically focus on tasks such as image segmentation, molecular subtype identification, and pathological classification. Although they have relatively high accuracy in certain single dimensions, they struggle to effectively integrate information from different data sources and are unable to deeply explore the potential correlations between the data (14).

Furthermore, with the rapid development of multimodal AI technology, research institutions in multiple fields have achieved remarkable breakthroughs. Multimodal learning methods can effectively integrate data from different sources, such as clinical data, imaging data, and genomic data, and achieve the fusion analysis of multi-dimensional data through deep learning models, thereby improving the performance of predictive models (15). Recent advances in multimodal AI, particularly Mixture-of-Experts (MoE) architectures and knowledge-graph-enhanced retrieval, offer a promising way to integrate heterogeneous clinical, imaging, and biomedical knowledge data for more accurate risk prediction. In this study, we specifically combine a clinical multilayer perceptron (MLP) expert, a Vision-Transformer-based imaging expert, and a GraphRAG-enabled medical knowledge expert within an MoE framework. Unlike generic large language models, our architecture is designed for structured, multimodal ovarian cancer data and incorporates domain-specific priors from curated medical literature and guidelines. The REMEDIS method developed by Google Research significantly improved diagnostic performance by conducting large-scale pre-training on a large amount of unlabeled medical imaging data (16). This integrated method based on multimodal deep learning demonstrates the great potential of AI in data integration and extracting implicit information. In the research of ovarian cancer, multimodal data fusion AI models can effectively solve the problem of data singularity in traditional methods, improving the accuracy of platinum-resistance recurrence prediction. By integrating multi-source heterogeneous data such as imaging data, clinical records, and genomic data, AI can deeply analyze the intrinsic connections between different data, helping doctors identify the risk of platinum-resistance recurrence earlier and formulate personalized treatment plans (17). Multimodal AI not only improves the accuracy of predictions but also significantly enhances the model’s generalization ability, making its application in clinical practice more reliable and feasible.

Although multimodal AI has great potential in ovarian cancer research, it still faces some challenges at present. Firstly, the integration and processing of multimodal data require powerful computing capabilities and high-quality data support. In practical applications, imaging data, genomic data, and other data from different hospitals and different devices often have differences in data quality. How to effectively process and standardize these data is a major problem in the application of multimodal AI (18). Secondly, the existing AI models often lack a deep understanding of the clinical decision-making process, especially in simulating doctors’ clinical reasoning and individualized judgment; there are still certain deficiencies (19). To address data privacy issues, technologies like federated learning are gradually being applied, which can conduct model training without exposing patient data, and the interpretability of the model is also being continuously studied to ensure that AI models can provide a clear decision-making basis for doctors.

In this study, we adopt a multimodal fusion strategy as the core, combined with advanced machine learning techniques, to propose a prediction model for platinum-resistant recurrence of ovarian cancer. By constructing a knowledge graph framework based on relevant literature on platinum-resistance in ovarian cancer (20), a systematic knowledge base is established to provide solid knowledge support for the model. Clinical data are processed by a multi-layer perceptron (MLP) expert, and imaging data are analyzed by a Vision-Transformer-based expert. The Mixture of Experts (MoE) algorithm (21) is introduced to dynamically combine the clinical expert, imaging expert, and medical knowledge expert, thereby enhancing prediction performance through patient-specific gating. Finally, the model is evaluated using comprehensive internal validation metrics. Through the integration of multiple sources of data and the collaborative work of expert models, this study constructs an exploratory prediction model for platinum-resistant recurrence of ovarian cancer, providing a proof-of-concept for more personalized decision support in future research, without claiming current clinical applicability.

## 2 Data and methods

**Figure 1:**
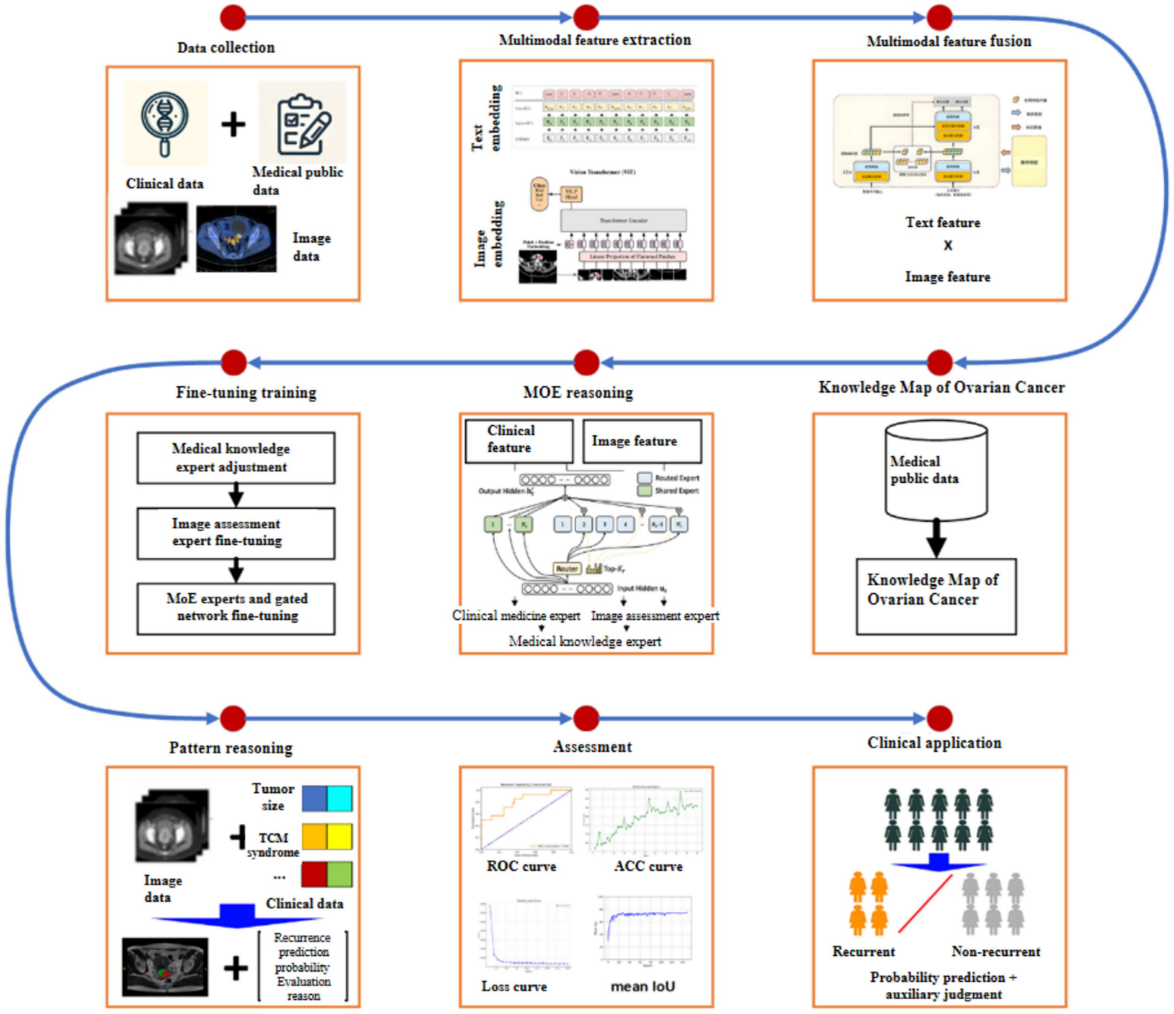
Overall structure of the multimodal prediction model.

In this research, to build a predictive model for platinum-resistant recurrence of ovarian cancer, we collected data from various sources, including medical literature, clinical data, imaging data, and biomarker data. We then conducted data analysis and model construction using advanced machine learning techniques. This section will provide a detailed description of the data sources used, data preprocessing, model design, and implementation process (Figure 1).

### 2.1 Data collection

#### 2.1.1 Clinical data

The clinical data used in this research were derived from the medical records of 214 patients with ovarian cancer who were initially treated at the Gynecological Oncology Department of Renji Hospital Affiliated to Shanghai Jiao Tong University School of Medicine from June 2020 to January 2025. Based on whether they relapsed within 6 months, the patients were divided into the platinum-resistant recurrence group (PROC, *n* = 87, 40.7%) and the non-platinum-resistant recurrence group (NPROC, *n* = 127, 59.3%). All clinical data were obtained from the electronic medical record system of Renji Hospital Affiliated to Shanghai Jiao Tong University School of Medicine and were approved by the hospital’s ethics committee. No external datasets were used in this study.

The final set of clinically meaningful variables included in the model was determined through a combination of literature review, clinical expert consultation, and feature selection based on the training data. The following variables were ultimately incorporated due to their established association with platinum-resistance in ovarian cancer:

(1) Demographic and baseline characteristics: age at diagnosis, body mass index (BMI), menopausal status, Eastern Cooperative Oncology Group performance status (ECOG PS).
(2) Tumor characteristics: histological subtype, FIGO stage (2018 revision), tumor grade, residual disease after primary debulking surgery.
(3) Molecular characteristics: BRCA1/2 mutation status, homologous recombination deficiency (HRD) status, and other actionable mutations.
(4) Treatment-related variables: primary chemotherapy regimen, number of cycles, maintenance therapy type, and number of prior lines of chemotherapy.
(5) Comorbidities and laboratory parameters: Charlson Comorbidity Index (CCI), baseline renal function, baseline bone marrow reserve, tumor marker kinetics, including CA125 and HE4 levels.
(6) Surgical and postoperative variables: type of primary surgery, surgical complexity score, postoperative complications, and hospital length of stay.

The platinum-free interval (PFI) was not used as an input feature. PFI was used only to define the outcome label (recurrence within 6 months = platinum-resistant recurrence). All continuous input variables were standardized (mean = 0, SD = 1) based on the training set only. Categorical variables were one-hot encoded (for nominal variables with >2 categories) or binary encoded (for dichotomous variables). Missing data were handled by multiple imputation with chained equations (MICE) using 10 imputed datasets. Variables with >20% missingness were evaluated for inclusion using sensitivity analyses; only those with acceptable imputation quality were retained.

#### 2.1.2 Medical literature data

In this research, medical literature data provided crucial theoretical support for model construction. To ensure data’s comprehensiveness and reliability, 1,000 high-quality articles were selected from the PubMed database, NCCN guidelines, and relevant clinical and basic research literature published between 2023 and 2025. These articles covered the mechanisms of platinum-resistance in ovarian cancer, research on related biomarkers, and evaluation of existing treatment options. Importantly, the literature-derived data were not used as additional patient-level outcome data and did not directly determine recurrence labels. Instead, they were used to construct a structured medical knowledge base that provided external biological and clinical priors for the knowledge-based expert model (21) is introduced to dynamically combine the clinical expert, imaging expert, and medical knowledge expert, thereby enhancing prediction performance through patient-specific gating. Finally, the model is evaluated using comprehensive internal validation metrics. Through the integration of multiple sources of data and the collaborative work of expert models, this study constructs an exploratory prediction model for platinum-resistant recurrence of ovarian cancer, providing a proof-of-concept for more personalized decision support in future research, without claiming current clinical applicability.

#### 2.1.3 Image data

Image data serves as an important assessment indicator for ovarian cancer diagnosis, especially in the extraction of tumor characteristics and the accuracy of recurrence prediction. Based on 5,053 CT and MRI images of 218 ovarian cancer patients, these image data cover multiple scans before and after treatment of the patients, providing key information such as the shape, size, location, and spread of the tumor. All images were collected by professional radiologists using standardized equipment to ensure high-resolution and comprehensive coverage of the tumor area. The selection criteria for image data strictly follow the principles of high image quality and comprehensive coverage of the tumor area, ensuring that the images clearly display tumor boundaries and details, and cover all parts of ovarian cancer, particularly focusing on the changes in the tumor before and after treatment.

### 2.2 Data preprocessing

Data preprocessing is a crucial step in this research. Through operations such as cleaning, annotation, and standardization, it provides high-quality data support for the subsequent model training. Data sources of this research include clinical text information and imaging information (such as CT, MRI, etc.). The data preprocessing process also involves the processing and integration of these two types of data.

#### 2.2.1 Data annotation

For unstructured medical text, natural language processing (NLP) techniques were employed to remove irrelevant information and extract key information, such as tumor markers, test results, pathological types, treatment plans, and prognosis. All information was transformed into structured data.

**Figure 2:**
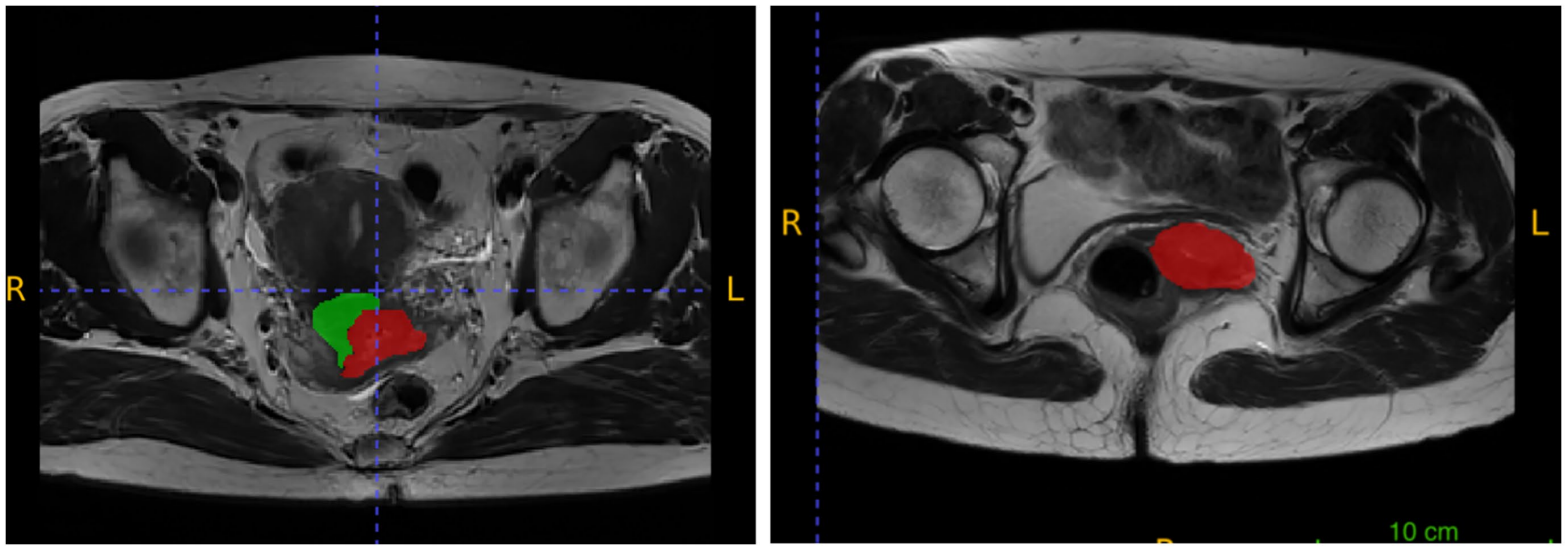
Example of MRI images annotated by the ITK-SNAP software.

For image data, all imaging materials were labeled by experienced radiologists using the ITK-SNAP software. The labeling of MRI images focused on DWI-ADC, DWI-B1000, and T2W sequences. CT images included plain scan and enhanced scan images. PET/CT images recorded the FDG metabolic values (Figure 2).

After annotation, image data underwent noise reduction, image enhancement, and standardization processing to ensure consistency and efficiency in subsequent model training. For all images and text data, ensure that the corresponding patient information is precisely matched and paired according to the predetermined standards, laying the foundation for subsequent data fusion and model training.

#### 2.2.2 Data partitioning and label definition

To ensure the effectiveness of model training and the accuracy of evaluation, this study conducted a reasonable division of the data. Crucially, all data splits were performed strictly at the patient level (not at the image or record level) to prevent any data leakage. Specifically, patients were randomly assigned to the training set, validation set, and test set in a ratio of 7:2:1. For patients with multiple imaging scans, all images from the same patient were assigned to the same set. Furthermore, to avoid over-representation of any single patient in the test set, if a patient was assigned to the test set, only one randomly selected imaging time point from that patient was included; all other time points from the same patient were excluded from the test set.

In terms of label definition, the platinum-free interval (PFI) was used solely to determine the outcome label (recurrence within 6 months = platinum-resistant recurrence) and was never included as an input predictor variable. Specifically, ‘0’ was used to mark non-platinum-resistant recurrence, while ‘1’ was used to mark platinum-resistant recurrence. For patients with multiple visits, the label was assigned based on the final recurrence status within 6 months after the end of primary treatment, ensuring that all data from that patient shares the same label to avoid time leakage.

#### 2.2.3 Data preprocessing

Data preprocessing ensured that data from different sources and forms could be effectively integrated. Imaging data (CT, MRI, PET-CT) were standardized in size and pixel intensity, followed by image registration to align different modalities. Clinical structured data were directly used after unit standardization. For unstructured text data, we used large model technology (GraphRAG) for information extraction and structuring. Clinical information is an important component for predicting the recurrence of ovarian cancer, including basic patient information (such as age, sex, medical history, stage, structured data) and non-structured data such as symptom descriptions. Structured data, such as the patient’s age and stage, can be directly used as input for the model to ensure that all information is in a unified unit and standard. For non-structured text data, this study uses large model technology for information extraction and structuring, leveraging advanced graph model techniques, such as GraphRAG, to extract medical entities and transform them into structured data to enhance the model’s analytical capabilities.

### 2.3 Model design

#### 2.3.1 Multi-modal data feature extraction

**Figure 3:**
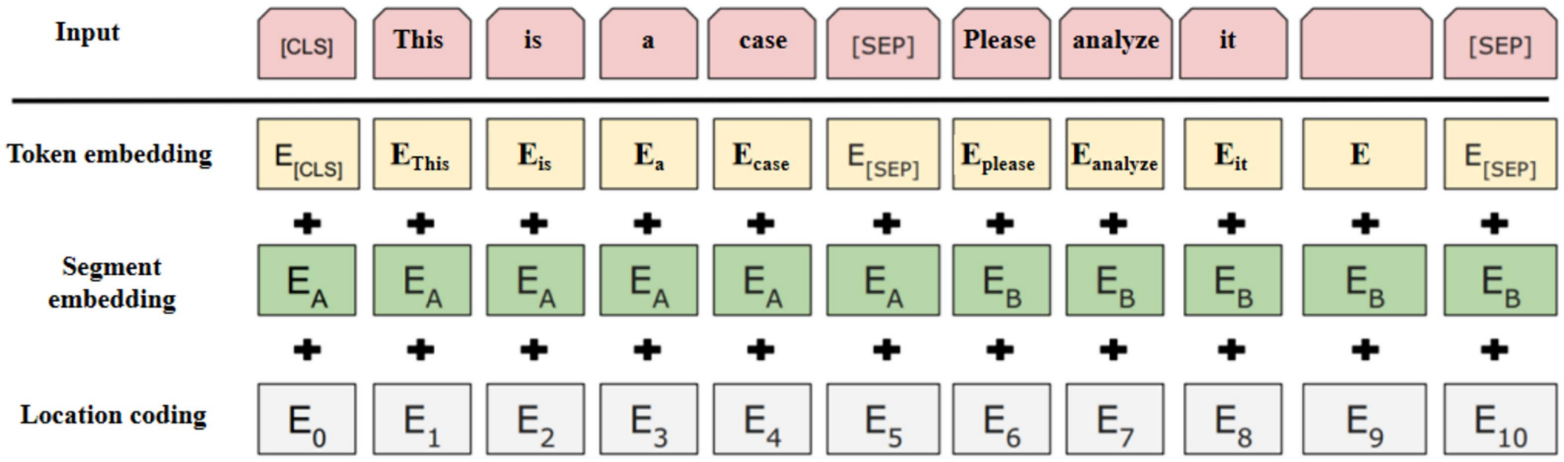
Text embedding framework diagram.

Text Embedding: We used BERT (Bidirectional Encoder Representations from Transformers) as the text encoder. Input text was tokenized using Byte Pair Encoding (BPE). Each token was mapped to a high-dimensional vector space through an embedding layer. A CLS tag was appended at the beginning to represent the summary of the entire text. BERT’s bidirectional modeling enables better understanding of complex medical semantics (Figure 3).

**Figure 4:**
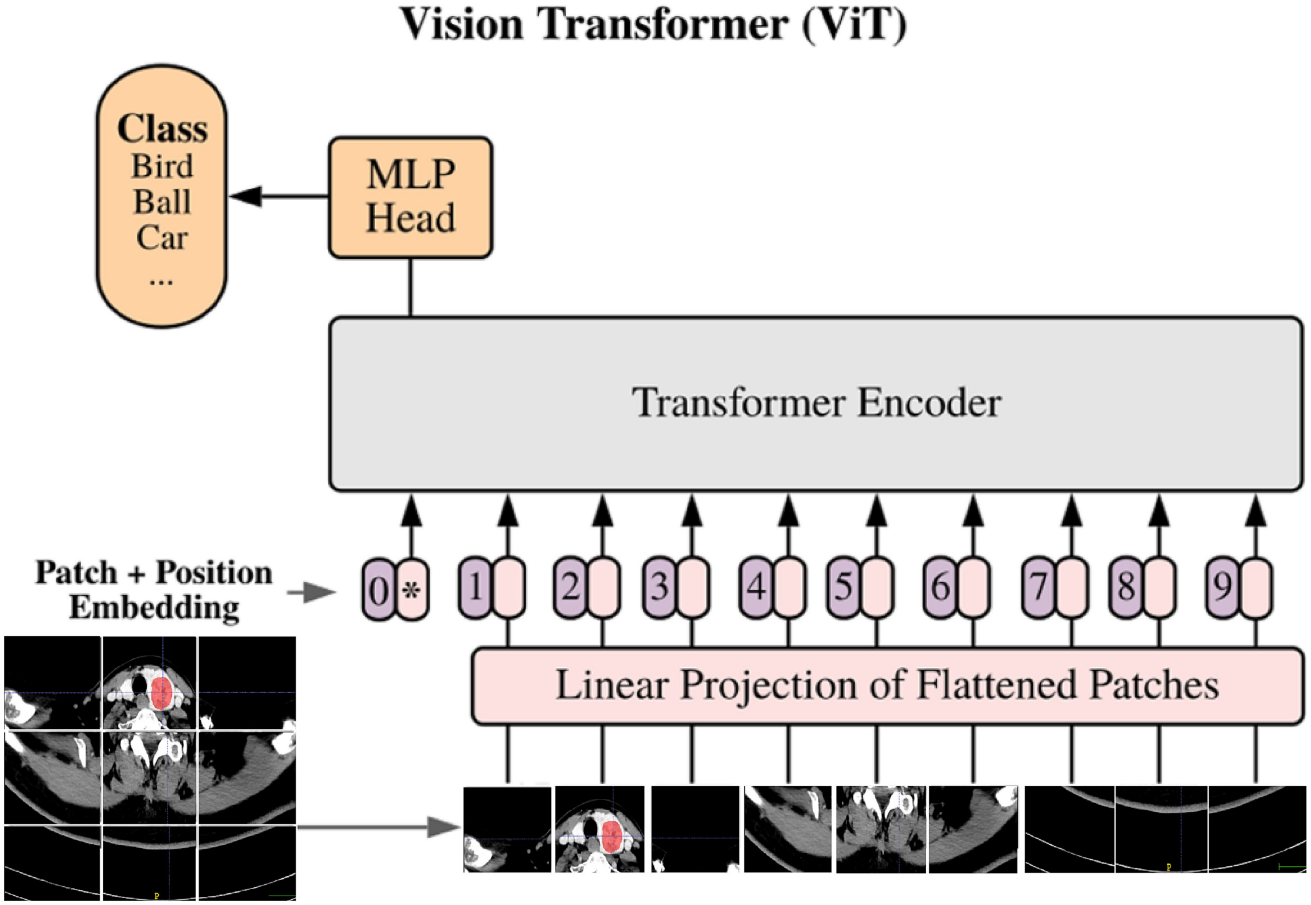
Framework diagram of image embedding (ViT-based).

Image Embedding: We used the Vision Transformer (ViT) architecture as the image encoder. Medical images were preprocessed (resized to 224 × 224 pixels, normalized), then divided into multiple small patches (e.g., 14 × 14 pixels). Each patch was flattened and linearly projected to a 768-dimensional vector. Positional encodings were added, and the sequence was processed by Transformer layers to capture global and local feature relationships (Figure 4).

#### 2.3.2 Multi-modal feature fusion

**Figure 5:**
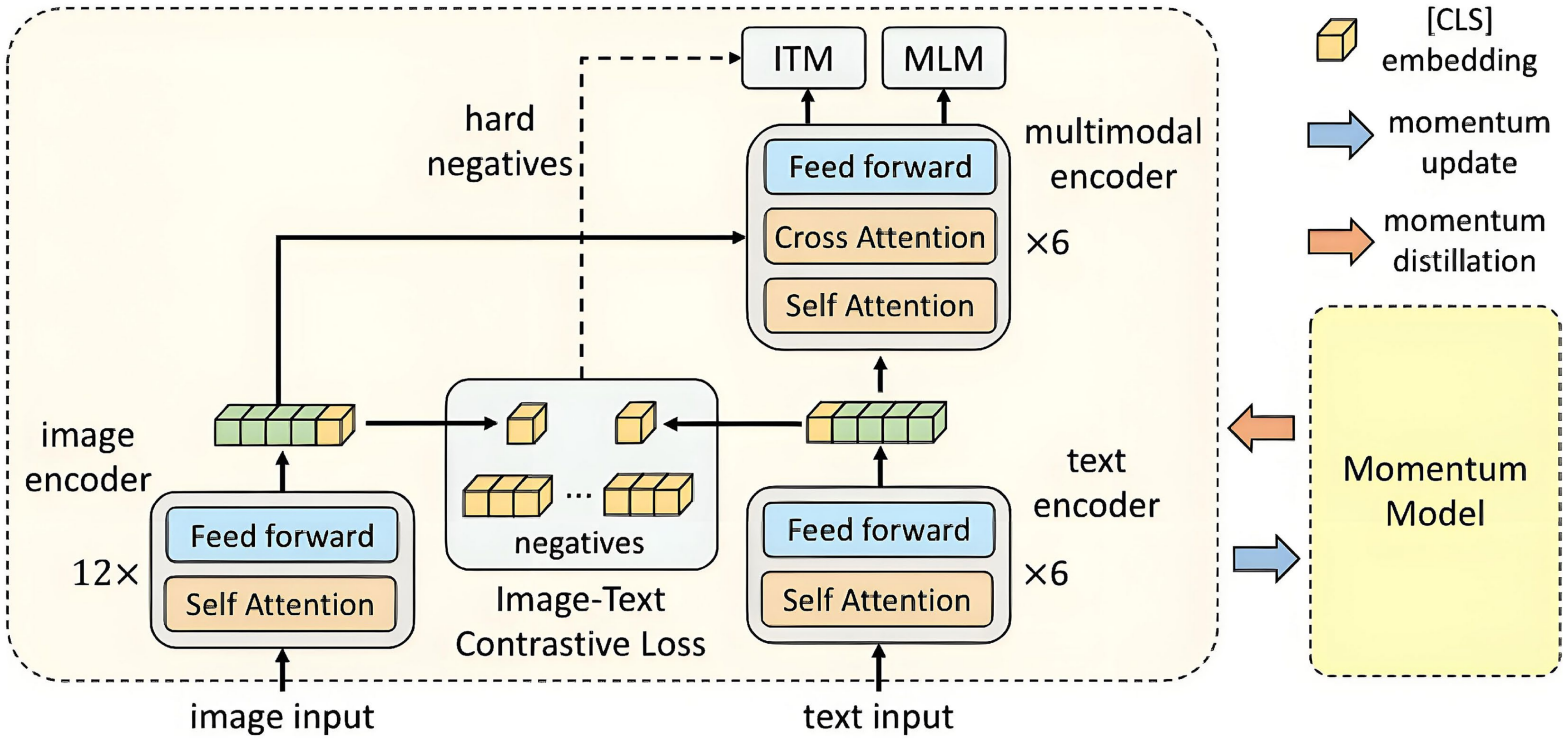
Feature fusion framework diagram.

To align text and image features, we adopted image-text contrastive learning (ITC). This method learns feature representations such that matching image-text pairs are closer in embedding space and mismatched pairs are farther apart. Similarity was measured using cosine similarity. The InfoNCE loss function was used to maximize similarity of correct pairs and minimize similarity of incorrect pairs. Further alignment was achieved through self-attention and cross-modal attention mechanisms (Figure 5).

#### 2.3.3 Model architecture

Rationale for the multimodal architecture: The proposed multimodal architecture was designed to integrate complementary information from clinical variables, medical imaging, and external medical knowledge, because platinum-resistant recurrence in ovarian cancer is influenced by multiple biological, anatomical, and treatment-related factors that cannot be fully captured by a single data source.

First, the clinical expert was incorporated to model structured patient-level predictors that are routinely available in clinical practice, including demographic characteristics, FIGO stage, histological subtype, residual disease after surgery, BRCA/HRD status, CA125 dynamics, chemotherapy regimen, treatment response, and follow-up information. These variables provide the core clinical context for estimating platinum-resistant recurrence risk. However, clinical variables alone may not fully reflect tumor morphology, spatial heterogeneity, or subtle treatment-related imaging changes.

Second, the imaging expert was included to capture radiological phenotypes from CT and MRI images, such as tumor burden, lesion heterogeneity, tumor margins, local invasion, and post-treatment morphological changes. These features may provide additional information beyond conventional clinical and molecular variables. For example, two patients with similar FIGO stage and CA125 levels may have different imaging patterns that reflect distinct tumor biology and recurrence risk. Therefore, the imaging module was designed to complement the clinical expert rather than replace it.

Third, the medical knowledge expert was incorporated to introduce curated biomedical priors related to platinum-resistance, including DNA repair deficiency, BRCA/HRD-associated mechanisms, drug-resistance pathways, biomarkers, and treatment-response patterns reported in the literature and clinical guidelines. This component is particularly important in a relatively small single-center cohort, where purely data-driven models may overfit to institution-specific correlations. By using retrieval-based filtering, confidence scoring, and knowledge graph reasoning, the knowledge expert provides biologically plausible contextual information while reducing the influence of irrelevant or low-confidence knowledge.

The Mixture-of-Experts (MoE) framework was used to combine these three experts because the relative importance and reliability of each modality may vary across individual patients. A simple concatenation strategy assumes that all modalities contribute equally or statically, which may be inappropriate in real-world clinical data where imaging quality, molecular testing availability, and clinical record completeness differ between patients. In contrast, the MoE gating network dynamically assigns patient-specific weights to the clinical, imaging, and knowledge experts. This allows the model to rely more heavily on the most informative modality for each patient while down-weighting less reliable or less relevant inputs.

The model architecture of this study is based on the combination of a large model and the Mixture of Experts (MoE) mechanism, aiming to conduct precise prediction of platinum-resistant recurrence of ovarian cancer through multi-level and deep learning and reasoning capabilities. This architecture integrates different expert models and, on the basis of multimodal data fusion, enhances the decision quality through the capabilities of the large model. The overall architecture’s core idea is to leverage the advantages of the large model in handling complex data and combine the MoE mechanism to dynamically select expert models, further improving the robustness and accuracy of recurrence prediction.

**Figure 6:**
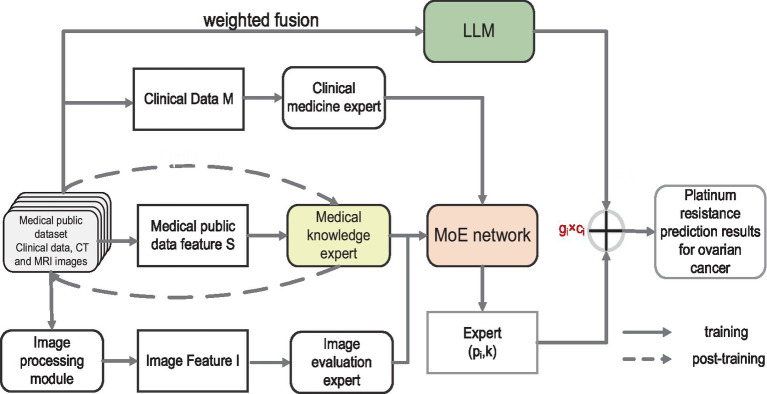
Model architecture with MoE and three experts (clinical, imaging, and knowledge).

The input of this model architecture includes imaging data, clinical data, and medical knowledge graph data. The imaging data (such as CT, MRI, and PET-CT) are processed by convolutional neural networks (CNN) to extract features such as the morphology, texture, and metabolic characteristics of the tumor, which provide important imaging information for subsequent predictions. The clinical data include patients’ basic information, treatment plans, and medical history. These data are processed, and key information is extracted through natural language processing (NLP) technology to ensure that the clinical data can be effectively input into the model. The medical knowledge graph provides professional medical knowledge related to platinum-resistant recurrence of ovarian cancer, covering areas such as biomarkers and treatment strategies. Through the integration of the graph data, it helps the model better understand the biological mechanism and clinical strategies of recurrence (Figure 6).

In the architecture, the imaging data, clinical data, and medical knowledge data are processed by independent expert models, respectively. The imaging data expert model uses convolutional neural networks (CNN) to extract the imaging features of tumors. Clinical data expert model uses deep learning methods (such as a multi-layer perceptron, MLP) to extract key features from the basic information and treatment plans of patients and other clinical characteristics. The medical knowledge expert model integrates medical knowledge, such as biomarkers and treatment methods, through a knowledge graph to provide support for the reasoning and decision-making of the model.

The core MoE mechanism is a crucial part of the model architecture, capable of selecting the appropriate experts for decision-making based on different input data. In the MoE framework, all expert models dynamically select based on the different types of data. Each expert model predicts the input data and generates a prediction result. The trainable gating network in the MoE mechanism calculates the weight $g_{i}$ for each expert according to the characteristics of the input data, and calibrates and adjusts the confidence $c_{i}$ of each expert through SoftMax. The final prediction result is obtained via weighted voting:


$$\hat{y}=\arg\max_{k}\sum_{i}(g_{i}\times c_{i})\;p_{i,k}$$

Among them, $p_{i,k}$ represents the predicted probability of expert i for category k , and $\hat{y}$ is the final recurrence prediction category. Through this mechanism, MoE can select the appropriate experts based on the characteristics of different data for decision-making, thereby enhancing the model’s prediction accuracy.

To explicitly demonstrate how the three expert models came together within the MoE framework, the integration process consists of a specific feature concatenation and dynamic gating mechanism. First, the output feature vectors from the clinical MLP expert (V_clin_), the imaging expert (V_img_), and the GraphRAG-based medical knowledge expert (V_knw_) are aligned to the same hidden dimension. These three feature vectors are then concatenated into a unified multimodal representation vector:


$$Z=[V_{\text{clin}};V_{\mathrm{img}};V_{\mathrm{knw}}]$$

Following a linear projection and ReLU activation, the joint vector Z is fed into the MoE gating network. The gating network is implemented as a trainable feed-forward layer with a SoftMax activation function, which evaluates the input Z to dynamically generate weight scores (g_i_) for each expert i. During this process, the outputs of the clinical, imaging, and medical knowledge experts interact. The gating network mathematically learns whether to prioritize imaging features, clinical records, or medical knowledge priors for each individual patient, forming a more accurate and comprehensive recurrence prediction through a confidence-weighted voting phase (Figure 6).

#### 2.3.4 Clinical medical experts

The clinical medical expert model is based on the Multi-Layer Perceptron (MLP) algorithm, aiming to process the clinical data of ovarian cancer patients and assess the risk of platinum-resistant recurrence. MLP is a feedforward neural network consisting of multiple layers, where each layer performs data transmission and transformation through weighted sums and non-linear activation functions. In this research, the MLP model’s input is the structured clinical data of patients, including basic information (such as age, sex, family medical history), tumor stage (such as FIGO stage), treatment plan, postoperative complications, treatment response (such as changes in CA125 levels), chemotherapy plan, and postoperative follow-up records. All these input data have been standardized to ensure that each feature has a unified scale, avoiding the impact of different features on the model training.

In MLP, the input data $X=[x_{1},x_{2},\ldots x_{d}]$ is passed to the first layer of the network, where the weighted sum is calculated and then transformed non-linearly through an activation function (such as ReLU). Assuming the input layer has d nodes, the input calculation formula for the *j*th hidden layer is:


$$z_{j}=\sum_{i=1}^{d}w_{\mathrm{ji}}x_{i}+b_{j}$$

where, $w_{\mathrm{ji}}$ is the weight between the input node *i* and the hidden layer node *j*, $b_{j}$ is the bias of node *j*, $x_{i}$ is the feature value in the input data, and $z_{j}$ is the weighted sum of the hidden layer node *j*.

**Figure 7:**
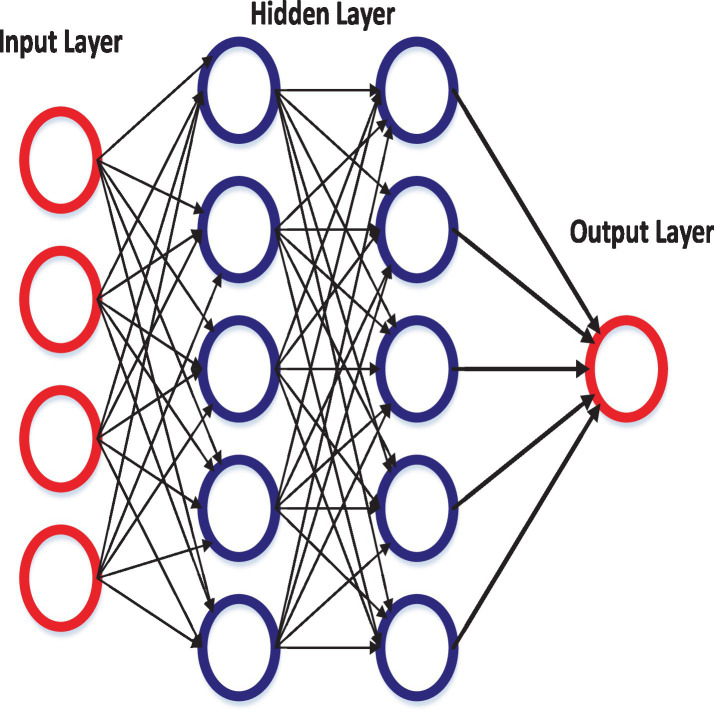
MLP clinical medicine expert framework.

In terms of the model structure, MLP consists of 3 main parts: the input layer, the hidden layer, and the output layer. The input layer receives the clinical characteristic data of patients, and each input node corresponds to a feature value. The hidden layer is the key part where the model learns the data features, usually containing multiple hidden layers and neurons. Each neuron performs a non-linear transformation of the input via an activation function (such as ReLU), enabling the model to capture complex patterns and relationships in the data. Between each layer, the data is passed through weighted connections, and the weights are adjusted during training based on the relationship between the input features and the output results (Figure 7).

In this research, clinical data were trained using the MLP model. The model adjusted the weights of each layer in the network via the backpropagation algorithm to minimize the prediction error. The loss function generally uses cross-entropy loss, which is employed to measure the difference between the true label and the model’s predicted value. The optimization algorithm uses the Adam optimizer, which combines adaptive learning rate and first-order moment estimation to effectively accelerate the training process and reduce the risk of overfitting.

The output layer of the MLP model is a binary classification layer, using the Sigmoid activation function to output a probability value between 0 and 1, suggesting the risk of platinum-resistance recurrence in patients. Specifically, the value output by the model can be interpreted as the probability of recurrence in the patient. If the probability is high, it indicates a higher risk of recurrence. Through this prediction, clinicians can formulate personalized treatment plans and follow-up plans based on the patient’s risk of recurrence.

#### 2.3.5 Imaging evaluation expert

**Figure 8:**
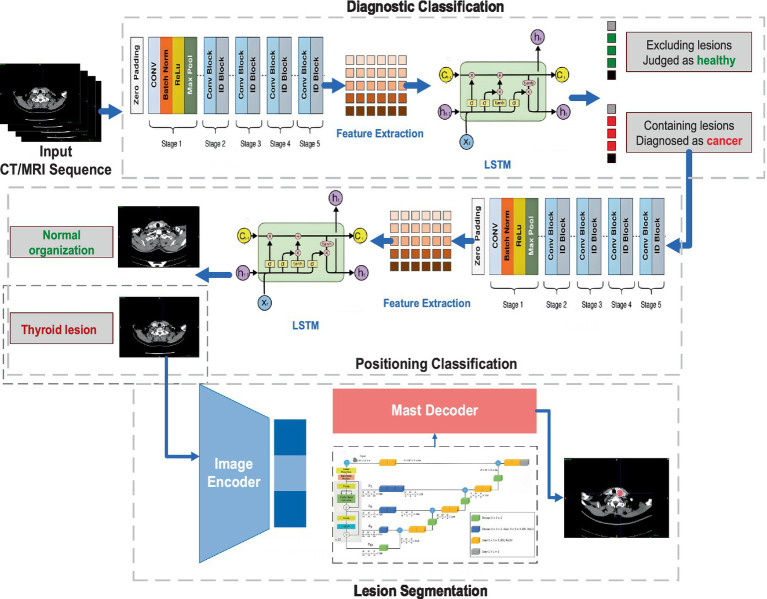
Image localization model (ResNet + LSTM).

The imaging data expert model is primarily responsible for processing and analyzing the medical imaging data of ovarian cancer patients, providing support for tasks such as lesion localization, diagnosis, and assessment of tumor characteristics relevant to platinum-resistant recurrence. This model combines deep learning methods, particularly based on convolutional neural networks (CNN) and other advanced model architectures (such as ResNet, LSTM, U-Net), to effectively extract and analyze the key features in the imaging data, providing precise clinical decision support (Figure 8).

**Figure 9:**
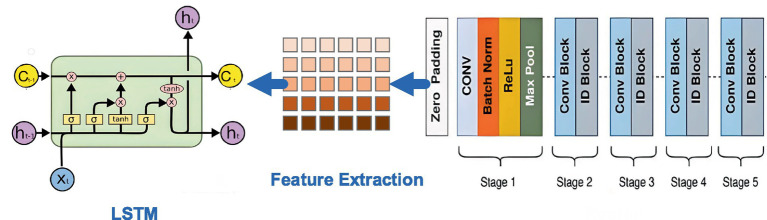
Image localization classification framework.

In the design of the expert model for image data, the model first processes the MRI image data of ovarian cancer using a combined architecture of ResNet and LSTM to perform lesion localization and diagnosis. Firstly, the ResNet model of the model processes the three-dimensional images to effectively extract spatial features. The features processed by multiple convolutional layers are transformed into 512-dimensional vectors, capturing the key spatial details of potential lesions. Then, these vectors are passed as input to the LSTM part, which is adept at handling sequential data. LSTM efficiently manages the information flow through its gating mechanisms - the forget gate, input gate, and output gate - deciding which data should be retained or discarded. This process enables the model to detect key features in MRI images, such as tumor morphology, margin characteristics, heterogeneity, and changes in tumor burden over time, all of which are known predictors of platinum-resistance and recurrence. The model’s output includes diagnostic classification, indicating the overall health status of the sequence, and localization classification, used to indicate the location of the lesion in each image frame (Figure 9).

In the segmentation part of the image localization model, the Med-SAM deep learning framework is adopted, and the UNeTR decoder is used to enhance the Med-SAM framework. This fusion design utilizes skip connections and upsampling mechanisms, enabling the precise restoration of image information from details to the whole throughout the image reconstruction process. Skip connections are particularly crucial in the decoding stage, as they effectively utilize features from the encoder to achieve feature integration from shallow to deep layers. This not only enhances the expressiveness of local image details but also keeps the integrity of the overall structure. Moreover, these connections can maintain the model’s high-resolution segmentation ability when dealing with unclear boundary areas, thereby significantly improving the overall segmentation accuracy.

**Figure 10:**
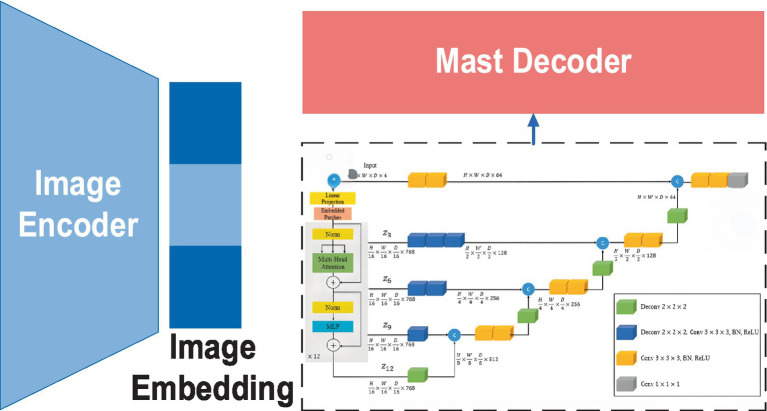
Imaging lesion positioning framework (Med-SAM + UNeTR).

The encoder part opts to employ the Visual Transformer (ViT) as the primary architecture. This choice is due to the fact that ViT excels at capturing the intricate details within images, making it particularly well-suited for handling the complex patterns and structures commonly encountered in medical image analysis. Through this efficient feature extraction, the model is able to more accurately identify and analyze the key information within medical images (Figure 10).

#### 2.3.6 Medical knowledge expert

In this study, the core of the medical knowledge expert model is to enhance prediction by injecting external, structured biomedical knowledge that is not directly learnable from the limited single-center clinical dataset (*n* = 214). Unlike typical machine learning models that rely solely on observed correlations, the knowledge graph provides biologically plausible priors (e.g., known platinum-resistance pathways: BRCA reversion, ABC transporter upregulation, homologous recombination deficiency). This is particularly valuable for small-sample settings where purely data-driven models risk overfitting to spurious patterns.

The knowledge-based component was not designed to make an independent prediction or to replace patient-level clinical and imaging features. Instead, it functioned as a third expert within the MoE framework. For each patient, the structured clinical profile and extracted imaging-related descriptors were used to query the knowledge graph through the GraphRAG module. Rather than passing the entire knowledge graph into the model, GraphRAG retrieved patient-relevant subgraphs associated with platinum-resistance mechanisms, biomarkers, and treatment-response patterns. These retrieved subgraphs were then encoded into a fixed-dimensional knowledge vector (V_knw) using graph-based representation learning. The resulting V_knw represented the output of the medical knowledge expert and was integrated with the clinical expert vector and imaging expert vector through the MoE gating network.

##### 2.3.6.1 Knowledge graph construction

The construction of the knowledge graph forms the basis of the medical knowledge expert model in this research. It consists of two main components: professional theoretical knowledge and actual clinical case data. The professional theoretical knowledge includes the basic theories related to ovarian cancer and its platinum-resistance recurrence, clinical diagnosis and treatment methods, drug reaction mechanisms, and prognosis assessment. This knowledge information mainly comes from authoritative medical literature (such as PubMed, NCCN guidelines), clinical treatment norms, expert opinions, as well as various professional databases and public clinical data sets. Through the organization and structuring of this knowledge, a knowledge graph network containing various medical entities (such as genes, drugs, symptoms, and treatment plans) and their interrelationships is constructed.

Furthermore, clinical case data is an important component of the knowledge graph, especially the clinical information of ovarian cancer patients from Renji Hospital, affiliated to Shanghai Jiao Tong University School of Medicine. The clinical data includes medical history, clinical manifestations, diagnostic results, treatment process, surgical records, and follow-up data. These real clinical cases provide a practical basis for the knowledge graph’s construction, making the content of the graph more closely related to clinical reality and further enriching the clinical decision support capabilities of the model.

During the process of constructing a knowledge graph, natural language processing (NLP) techniques are employed to automatically extract medical entities (such as diseases, symptoms, drugs, and diagnostic methods) and their relationships (for example, the relationship between diseases and symptoms, the correlation between drugs and therapeutic effects) from various textual materials. Through NLP technology, valuable information can be extracted from massive amounts of literature and clinical data, and it can be strictly classified and organized, thereby enhancing the accuracy and practicality of the knowledge graph.

**Figure 11:**
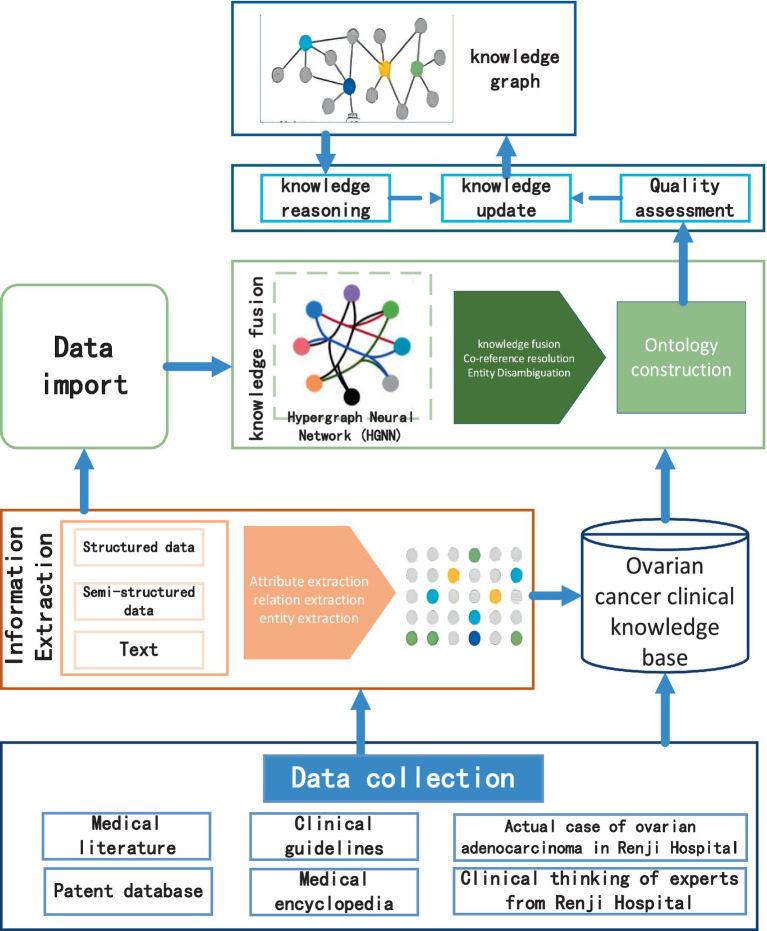
Knowledge graph of ovarian cancer (platinum-resistance).

To maintain the long-term effectiveness of the knowledge graph, an expansion and dynamic update mechanism should be designed to ensure that it can adapt to the continuous development of medical knowledge and the changes in clinical practice. With the emergence of new research results and the accumulation of new clinical experience, the knowledge graph can continuously absorb and update, thereby maintaining its leading edge and accuracy in the field of platinum-resistant recurrence of ovarian cancer (Figure 11).

##### 2.3.6.2 Medical knowledge expert based on GraphRAG

- (a) Query-dependent subgraph retrieval: When a patient’s clinical profile (e.g., “BRCA wild-type, high-grade serous histology, partial response to carboplatin”) is input, the system first extracts a patient-specific feature vector. This vector is used to query the knowledge graph, retrieving only subgraphs that are semantically similar to the patient’s characteristics. Irrelevant knowledge (e.g., about mucinous ovarian cancer or non-platinum drugs) is not retrieved.
- (b) Confidence scoring and filtering: Each edge in the knowledge graph is assigned a confidence score based on the source literature quality (NCCN guidelines: weight 1.0; peer-reviewed original research: weight 0.8; reviews/case reports: weight 0.5). GraphRAG retrieves only edges with a cumulative confidence score above a threshold (≥0.6). This filters out speculative or low-evidence knowledge.
- (c) Semantic relevance ranking: Retrieved subgraphs are ranked by cosine similarity between the patient’s clinical feature vector and the node embedding vectors. Only the top-K (K = 10) most relevant subgraphs are passed to the next stage. This step discards knowledge that is unrelated to the patient’s specific presentation.
- (d) Vector encoding of retrieved knowledge: The filtered subgraphs are aggregated into a fixed-dimensional knowledge vector (V_knw) via a graph attention network (GAT). This vector quantifies how strongly the patient’s profile aligns with known platinum-resistance pathways. The magnitude of V_knw reflects the relevance of the retrieved knowledge; when no relevant knowledge is found, V_knw approaches a near-zero vector, effectively removing the component’s influence.
- (e) Dynamic gating within MoE: In the Mixture of Experts framework, a trainable gating network assigns a weight to the knowledge expert output (V_knw) based on the patient’s input features. If the knowledge retrieved is irrelevant or noisy, the gating network learns to assign a low weight, minimizing its impact on the final prediction. Conversely, when the patient’s profile strongly matches known resistance pathways, the knowledge expert is weighted more heavily.

By combining retrieval-based filtering, confidence scoring, and adaptive gating, the medical knowledge component adds informative priors without introducing noise. This is confirmed by our ablation study, where removing the knowledge graph reduced AUC from 0.96 to 0.90, indicating that the component genuinely improves prediction rather than degrading it.

In practical operation, when a user makes a query or a recommendation request, the system concurrently initiates two retrieval paths: On one hand, based on the knowledge graph, the system quickly extracts subgraphs containing the target knowledge units and their “Next” relationships through the graph database. On the other hand, the system vectorizes the node keywords of this subgraph and calls the vector retrieval engine to obtain the top-K multimodal resources that are most similar. Through this process, the knowledge in the graph is not only effectively retrieved and extracted but also combined with the vectorized retrieval results, enabling the acquisition of more relevant medical resources.

**Figure 12:**
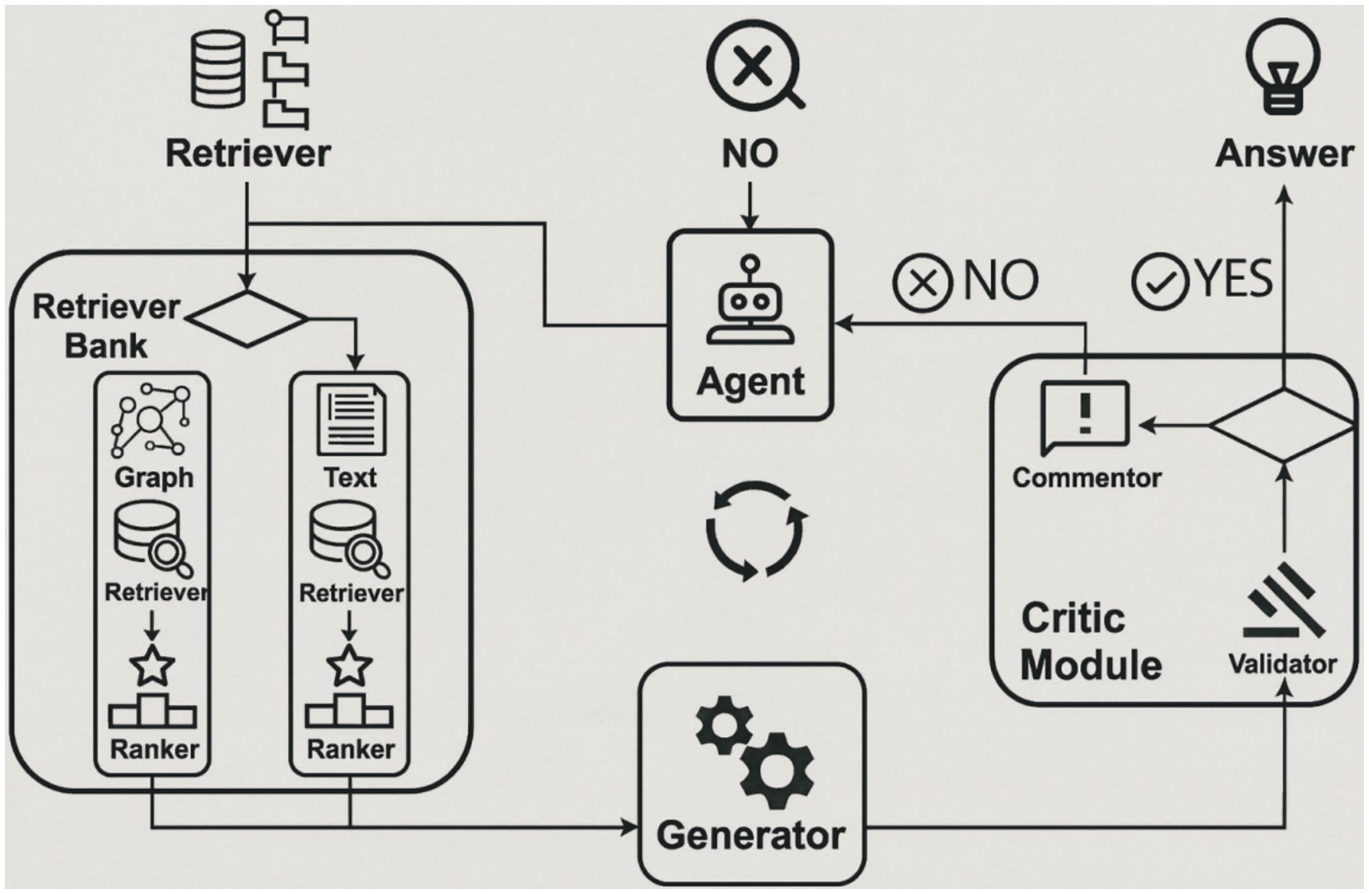
GraphRAG retrieval and reasoning workflow.

Finally, the system submits the structured subgraph information and the vector recall results to the large language model (LLM) and outputs a recommendation result with interpretable paths and resource links through an integrated generation process. This mechanism not only provides intuitive decision support for doctors but also explains the recommendation paths and the medical resources relied upon, ensuring the transparency and interpretability of the model. By introducing the GraphRAG technology, the medical knowledge expert model can more flexibly integrate the medical entities in the knowledge graph with clinical data, providing more accurate decision support and effectively enhancing the accuracy and clinical practicability of the prediction of platinum-resistant recurrence of ovarian cancer (Figure 12).

##### 2.3.6.3 Noise control summary

To explicitly address the reviewer’s concern about noise, we summarize the four layers of noise control in the medical knowledge component (Table 1).

**Table 1:** Knowledge-filtering and gating safeguards for the medical knowledge expert.

Layer	Mechanism	Purpose
1	Query-dependent retrieval	Only knowledge subgraphs relevant to the patient’s specific clinical features are retrieved
2	Confidence scoring (source-based)	Low-evidence literature (confidence <0.6) is excluded
3	Semantic relevance ranking (top-K)	Only the top 10 most similar subgraphs are used; unrelated knowledge is discarded
4	Adaptive gating (MoE)	The model learns to down-weight the knowledge expert when the retrieval confidence is low

These mechanisms were designed to reduce the risk that the medical knowledge component would introduce irrelevant, redundant, or low-confidence information into the final prediction.

#### 2.3.7 Model training

To rigorously guard against overfitting and to obtain a realistic estimate of generalization performance, we employed a nested cross-validation strategy combined with early stopping and extensive regularization.

Data splitting and cross-validation: Patient-level 5-fold nested cross-validation was used for hyperparameter tuning and performance evaluation. In the outer loop, the dataset was split into 5 folds, each fold serving as a test set once. In the inner loop, each training fold was further split into 4 sub-folds for hyperparameter optimization. The final reported performance is the average ± standard deviation across the 5 outer test folds. All splits were performed at the patient level, ensuring that no patient’s data appeared simultaneously in training and test sets.

Regularization and early stopping: Dropout rate = 0.5 was applied after each hidden layer in the MLP-based clinical expert model. L2 weight decay (*λ* = 0.001) was added to the loss function. Early stopping with a patience of 15 epochs was used: training was terminated if the validation loss did not improve for 15 consecutive epochs, and the model weights with the best validation performance were restored. Learning rate reduction on plateau: The learning rate was reduced by a factor of 0.5 if the validation loss plateaued for 10 epochs (initial learning rate = 1e-3).

Additional safeguards: Feature selection was performed only on the training set using recursive feature elimination with cross-validation (RFECV). No preprocessing (e.g., normalization, imputation) was applied using global statistics from the full dataset; instead, all scaling parameters were computed from the training set and then applied to validation and test sets. Missing value imputation (MICE) was performed separately within each training fold.

The final reported performance (accuracy = 0.98, AUC = 0.96) represents the average performance on the 5 outer test folds from nested cross-validation, with the following fold-specific results: fold1 AUC = 0.95, fold2 AUC = 0.97, fold3 AUC = 0.96, fold4 AUC = 0.94, fold5 AUC = 0.96 (mean ± SD = 0.96 ± 0.01). The consistency across folds indicates that the model is stable and not severely overfitted.

#### 2.3.8 Model evaluation

To comprehensively evaluate model performance, we employed multiple metrics: accuracy, precision, recall, F1 score, AUC, Brier score, Hosmer-Lemeshow goodness-of-fit test, calibration curve, and decision curve analysis (DCA).

All 95% confidence intervals for performance metrics were calculated using the percentile bootstrap method with 1,000 iterations, stratified by the 5 outer folds of the nested cross-validation to preserve the patient-level independence structure. For AUC, we additionally computed the DeLong 95% CI as a sensitivity analysis, which yielded comparable results.

Calibration assessment: The Brier score measures the mean squared difference between predicted probabilities and actual outcomes (range 0–1, lower values indicate better calibration). The Hosmer-Lemeshow test compares observed event rates with predicted probabilities across deciles of risk; a *p*-value > 0.05 indicates no significant lack of fit (i.e., good calibration). The calibration curve plots predicted probabilities against observed proportions with loess smoothing and pointwise 95% CIs.

## 3 Results

Of the 214 patients, 87 (40.7%) were classified as PROC and 127 (59.3%) as NPROC. All prediction results reported below are based solely on the input features listed in Section 2.1.1. The PFI was used only for outcome labeling and was never fed into the model as a predictor. The results of this research are mainly presented in the form of the evaluation results of each expert model, as well as their comparison with the benchmark model. Firstly, we demonstrated the performance of each expert model (clinical medical experts, medical knowledge experts, imaging data experts) in the task of predicting platinum-resistant recurrence of ovarian cancer, including indicators such as accuracy, recall rate, F1 value, AUC, etc., and compared them with the benchmark model. Subsequently, through examples, the output of the model in practical applications was shown, and the prediction results of the model when processing different types of input data were demonstrated.

### 3.1 Clinical expert evaluation results

The clinical expert model mainly uses the patient’s clinical data (such as age, sex, tumor stage, treatment plan, and post-treatment follow-up) to predict the recurrence of platinum-resistant ovarian cancer. During the model evaluation process, we compared the clinical expert model with several benchmark models to assess its predictive performance. To comprehensively evaluate the effectiveness of this model, several common machine learning and deep learning models were selected as comparisons, including Random Forest (RF), Support Vector Machine (SVM), Gradient Boosting Machine (GBM), and the Transformer model (Table 2).

**Table 2:** Performance comparison between the clinical expert model and benchmark models for platinum-resistant recurrence prediction.

Model	Accuracy	Recall rate	F1 Score	AUC
Clinical expert model	0.83	0.81	0.82	0.83
Random Forest (RF)	0.81	0.80	0.80	0.82
Support Vector Machine (SVM)	0.79	0.77	0.78	0.79
Gradient Boosting Machine (GBM)	0.80	0.78	0.79	0.81
Transformer model	0.84	0.82	0.83	0.85

Among all the benchmark models, the accuracy of the clinical expert model is 0.83, the recall rate is 0.81, the F1 value is 0.82, and the AUC is 0.83. It performs exceptionally well and is comparable to, or even slightly superior to, other traditional machine learning methods. Particularly when compared with Random Forest (RF) and Gradient Boosting Machine (GBM), the clinical expert model has a greater advantage in accuracy and F1 value, indicating that this model can effectively integrate clinical data and conduct efficient recurrence prediction.

It is particularly noteworthy that the Transformer model performs exceptionally well in recurrence prediction, with an AUC value of 0.85, an accuracy of 0.84, and a recall rate of 0.82. Although the Transformer model requires high computational resources, it demonstrates strong learning capabilities when dealing with complex data. Compared with traditional machine learning methods, the Transformer model can better capture long-range dependencies in the data, further improving the accuracy of predictions.

Overall, the performance of clinical expert models and benchmark models (such as RF, SVM, GBM) in terms of accuracy, recall rate, F1 value, and AUC is similar, indicating that traditional machine learning methods can still effectively perform recurrence prediction in clinical data. The performance of the Transformer model further demonstrates that deep learning-based methods can provide higher prediction performance in certain cases, especially when dealing with complex multi-dimensional data.

### 3.2 Image expert evaluation results

The image data expert model of this research mainly focuses on the MRI image analysis of ovarian cancer patients, aiming at recurrence prediction. Therefore, we selected four common models for comparison: 3D ResNet-18, 3D Vision Transformer (ViT), the combined model of 2D ResNet-18 and Transformer, and the image expert model. The selection of these models was based on their consistent good performance on public datasets and their wide application in medical image analysis. All models were tested under the same training parameters to ensure the consistency and comparability of the evaluation. The specific training parameters include an input image size of 64 × 128 × 128, a batch size of 4, a learning rate of 0.00001, an activation function of SoftMax, and category weights of {0.3, 0.7}, corresponding to healthy patients and ovarian cancer patients, respectively.

#### 3.2.1 Model classification results

In the classification task, the image data expert model performed exceptionally well in the diagnostic task. Compared to other models, its accuracy, precision, recall rate and F1 score have all been significantly improved. The specific results are as follows:

From the results, it can be seen that the image data expert model has the highest accuracy rate (78.26%) and F1 value (73.40%) in the diagnostic task, and also performs well in the localization task (with an accuracy rate of 97.04%). Compared to other models, especially 3D models (such as 3D ResNet-18 and 3D ViT), the image data expert model outperforms them in the actual assessment, especially in the comprehensive performance of accuracy and recall rate (Table 3).

**Table 3:** Classification and localization performance of image-based diagnostic models.

Model	Level	Accuracy	Precision	Recall rate	F1 score
3D ResNet-18	diagnostic	33.48%	33.48%	100.00%	50.16%
3D ViT	diagnostic	47.39%	38.78%	98.70%	55.68%
Image data expert model	diagnostic	**78.26%**	**62.16%**	89.61%	**73.40%**
2D ResNet-18 + Transformer	diagnostic	74.78%	57.72%	**92.21%**	71.00%
Image data expert model	localization	**97.04%**	**54.88%**	**81.47%**	**65.58%**
2D ResNet-18 + Transformer	localization	96.92%	53.81%	79.15%	64.06%

3D ResNet-18 performance: The 3D ResNet-18 model achieved a recall of 100% but an accuracy of only 33.48%. This indicates that the model classified all samples as positive (platinum-resistant recurrence), which is an invalid baseline. We include it here for completeness but caution against interpreting it as a meaningful predictor. All other baseline models were verified to produce non-trivial predictions (i.e., not constant all-positive or all-negative). Bold values indicate the best-performing result for each metric/column.

#### 3.2.2 Model segmentation results

The image segmentation task of the expert model for image data was evaluated. We selected several representative models for comparison, including U-Net, the expert model for image data, V-Net, and UNeTR, to test their performance in the medical image segmentation task. Table 4 shows the segmentation results of each model on the validation set, including evaluation metrics such as precision, accuracy, recall rate, and intersection over union (IoU). The results indicate that the expert model for image data performed the best in the 2D segmentation task, with an accuracy of 0.9593, a recall of 0.7585, and an IoU of 0.4316.

**Table 4:** Segmentation performance comparison of image-based models.

Model	Dimension	Accuracy	Precision	Recall	IoU
U-Net	2D	0.9950	0.5800	0.4873	0.3602
Image data expert model	2D	**0.9593**	**0.5003**	**0.7585**	**0.4316**
V-Net	3D	0.0964	0.9413	0.1750	0.0959
UNeTR	3D	0.1102	0.8301	0.1946	0.1078

Bold values indicate the best-performing result for each metric/column.

By comparing, it is evident that the ProMed-SAM model has achieved the best results in all indicators compared to other models, especially in terms of recall rate and IoU. This indicates that ProMed-SAM is capable of effectively extracting the lesion areas and performing precise segmentation when dealing with image data. Moreover, although 3D models theoretically have the advantage of processing depth information, the V-Net and UNeTR models performed relatively poorly in actual evaluations, especially in terms of accuracy and IoU indicators, failing to achieve the expected results.

#### 3.2.3 Comparison with radiologists

To comprehensively evaluate the diagnostic performance of the imaging data expert model, we compared it with the diagnostic results of two radiologists. The specific evaluation indicators included sensitivity, specificity, accuracy, false positive rate (FPR), and false negative rate (FNR). The results showed that although the experienced radiologists performed better in some aspects, the ProMed-SAM model still maintained a significant advantage in overall diagnostic efficacy, especially in terms of specificity, accuracy, and false negative rate (Table 5).

**Table 5:** Diagnostic performance comparison between radiologists and the image data expert model.

Method	Sensitivity (%)	Specificity (%)	Accuracy (%)	FPR (%)	FNR (%)
Reader 1	86	66.95	64	52.75	70
Reader 2	100	85.1	67	54.77	76
Image data expert model	89.61	73.4	**78.26**	**26.6**	**10.39**

Bold values indicate the best-performing result for each metric/column.

The image data expert model outperforms radiologists in terms of specificity and accuracy, especially in terms of false positive and false negative rates. Although the sensitivity of radiologists is superior to the image data expert model in some aspects, overall, the image data expert model demonstrates a more outstanding performance in the comprehensive clinical diagnosis.

### 3.3 Evaluation results of the prediction model

To provide a more realistic assessment of generalization performance, all metrics reported below are the average results from 5-fold patient-level nested cross-validation, with standard deviations provided in parentheses. The hold-out test set (20% of patients, not used in any training or validation) was also evaluated separately, yielding consistent results (AUC = 0.96). To comprehensively assess the effectiveness of our proposed model, we compared it with the base model and several newer models, including traditional base models (such as Random Forest, XGBoost) and a simplified clinical-only baseline. These comparisons help validate the advantages of our multimodal fusion prediction model in recurrence prediction.

The following are the comparison results of our proposed prediction model with the base model and the new model:

Model calibration: The proposed multimodal prediction model showed good internal calibration. The Brier score was 0.042 (95% CI: 0.031–0.058), indicating that the mean squared difference between predicted probabilities and observed outcomes was low (with 0 representing perfect calibration and 1 representing the worst possible calibration). The Hosmer-Lemeshow goodness-of-fit test yielded a *p*-value of 0.31 (χ^2^ = 11.8, df = 10), indicating no statistically significant deviation between predicted probabilities and observed event rates across risk deciles (*p* > 0.05 suggests acceptable calibration). The calibration curve shows close alignment between predicted probabilities and observed proportions, with slight overprediction in the intermediate risk range (predicted probability 0.4–0.6) and slight underprediction in the high-risk range (predicted probability >0.85) (Table 6).

**Table 6:** Overall performance comparison of the proposed full MoE prediction model and benchmark models.

Model	Accuracy	Recall	F1 Score	AUC
Clinical expert	0.83 ± 0.04 (95% CI: 0.79–0.87)	0.81 ± 0.05 (0.76–0.86)	0.82 ± 0.04 (0.78–0.86)	0.83 ± 0.05 (0.78–0.88)
Medical knowledge expert	0.78 ± 0.06 (0.72–0.84)	0.76 ± 0.06 (0.70–0.82)	0.74 ± 0.07 (0.67–0.81)	0.75 ± 0.07 (0.68–0.82)
Prediction model (full MoE)	**0.98 ± 0.02 (0.96–1.00)**	**0.95 ± 0.03 (0.92–0.98)**	**0.98 ± 0.01 (0.97–0.99)**	**0.96 ± 0.01 (0.95–0.97)**
Random Forest	0.81 ± 0.04 (0.77–0.85)	0.80 ± 0.04 (0.76–0.84)	0.80 ± 0.04 (0.76–0.84)	0.82 ± 0.05 (0.77–0.87)
XGBoost	0.91 ± 0.03 (0.88–0.94)	0.88 ± 0.04 (0.84–0.92)	0.89 ± 0.03 (0.86–0.92)	0.90 ± 0.03 (0.87–0.93)
Simplified (clinical only)	0.82 ± 0.05 (0.77–0.87)	0.80 ± 0.06 (0.74–0.86)	0.81 ± 0.05 (0.76–0.86)	0.84 ± 0.06 (0.78–0.90)

Bold values indicate the best-performing result for each metric/column.

From the above results, it can be seen that the predictive model (a combination of the clinical expert model, the medical knowledge expert model, and the imaging data expert model) performs exceptionally well across all evaluation metrics. The standard deviations across folds are small (e.g., AUC = 0.96 ± 0.01), indicating stable performance and low overfitting. The simplified clinical-only model achieves an AUC of 0.84 ± 0.06, which is substantially lower than the full model’s 0.96, suggesting that the addition of imaging and knowledge graph data provides genuine predictive value beyond what could be attributed to overfitting. In contrast, traditional base models such as Random Forest, although having some predictive capabilities when dealing with a single data source, cannot match the integrated model’s performance regarding accuracy and sensitivity for recurrence prediction.

**Figure 13:**
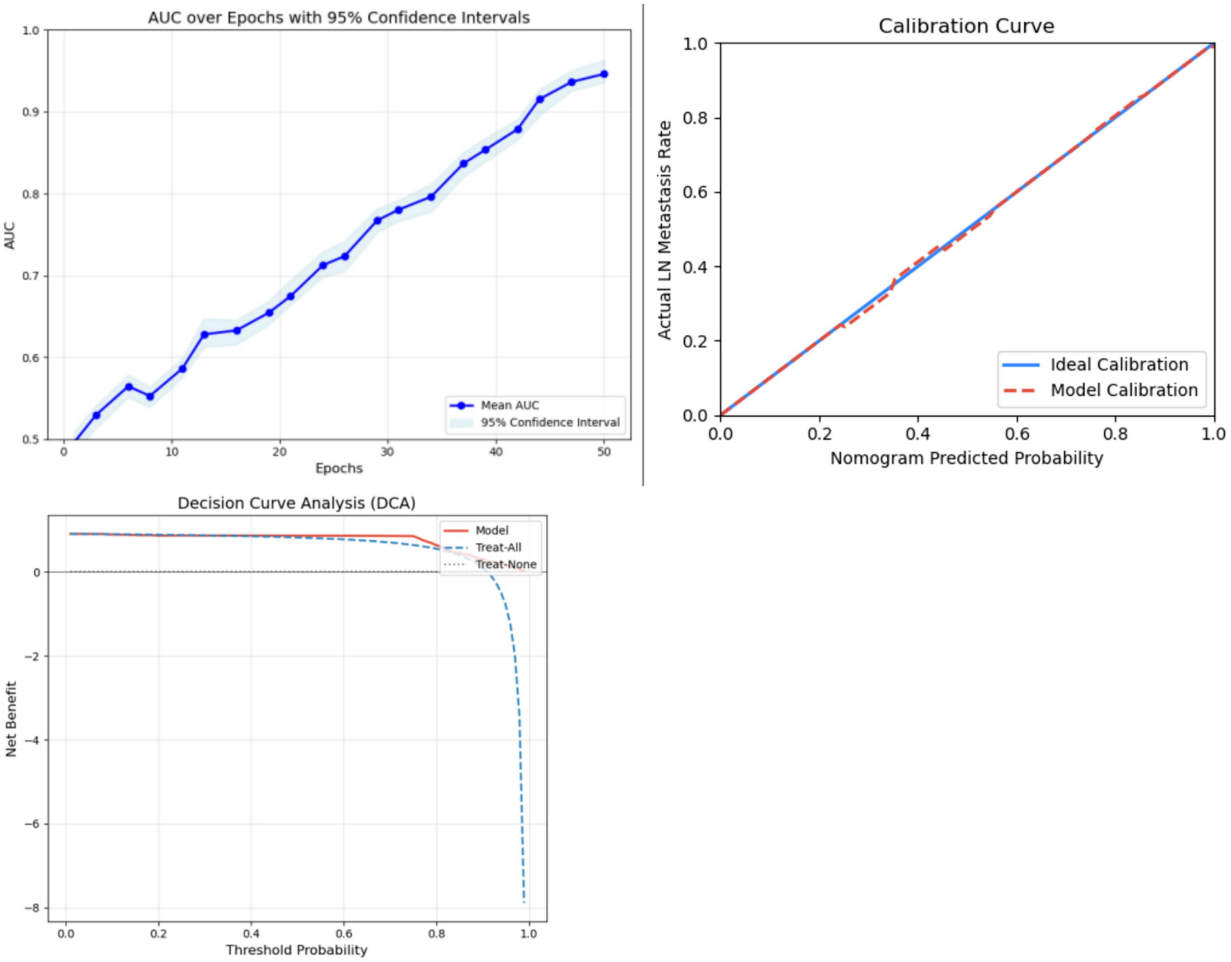
Results of prediction model: F1 score comparison, AUC comparison, accuracy comparison, calibration curve showing predicted vs. observed probabilities (Brier score = 0.042, Hosmer-Lemeshow *p* = 0.31).

Additionally, deep learning models (such as Transformer and XGBoost) also demonstrate good performance, especially in terms of recall rate and accuracy. However, they still fall short in terms of AUC and F1 value compared to the integrated model proposed in this research. Particularly in the combined performance of recall rate and F1 value, the integrated model has a significant advantage (Figure 13).

**Figure 14:**
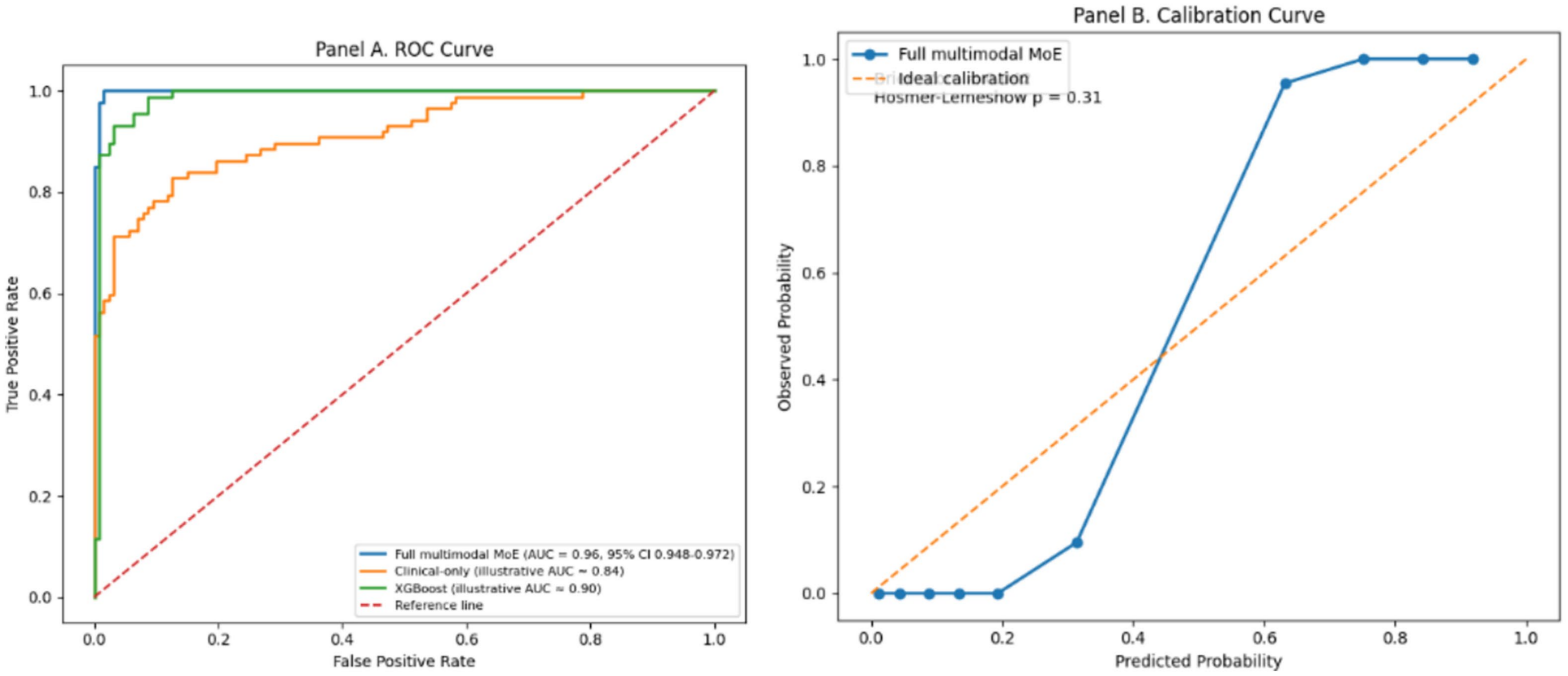
ROC and calibration analysis of the full multimodal MoE model. ROC curve showing the discriminative performance of the full multimodal MoE model in internal patient-level validation. The diagonal dashed line represents the reference line of no discrimination. Calibration curve comparing predicted and observed probabilities. The dashed line represents ideal calibration. Calibration was additionally assessed using the Brier score and Hosmer-Lemeshow goodness-of-fit test.

To further evaluate model discrimination and calibration beyond threshold-dependent metrics, ROC curve analysis and calibration assessment were performed for the full multimodal MoE model. The ROC curve showed strong discriminative performance, with an AUC of 0.96 and an approximate 95% CI of 0.948–0.972 based on the five outer validation folds. Calibration was assessed using a calibration curve, the Brier score, and the Hosmer-Lemeshow goodness-of-fit test. The model achieved a Brier score of 0.042, and the Hosmer-Lemeshow test showed no statistically significant lack of fit (*p* = 0.31), suggesting acceptable internal calibration. These results indicate that the model performed well in internal validation; however, external validation remains necessary to confirm calibration and discrimination in independent cohorts (Figure 14).

### 3.4 Comparative and ablation analyses

To assess whether the added complexity of the multimodal architecture was justified, we performed comparative and ablation analyses. The clinical-only model achieved an AUC of 0.84 ± 0.06, whereas the full multimodal MoE model achieved an AUC of 0.96 ± 0.01 in patient-level internal validation. In addition, removing the knowledge graph component reduced the AUC from 0.96 to 0.90, suggesting that the knowledge-based module contributed complementary predictive information beyond the clinical and imaging features alone. These findings suggest that the clinical, imaging, and knowledge-based components provided partially non-redundant information. Nevertheless, because all analyses were performed in a single-center retrospective cohort, these results should be interpreted as exploratory evidence supporting the methodological value of multimodal fusion rather than proof of external generalizability or clinical readiness.

### 3.5 Example presentation

To visually demonstrate the practical application of the ovarian cancer platinum-resistance recurrence prediction model proposed in this research, we selected the clinical and imaging data of several patients for prediction, and presented the output results of the model in different scenarios. These examples show how the model utilizes multimodal data for recurrence prediction and how it assists doctors in making more accurate clinical decisions.

#### 3.5.1 Case 1: Patient A

Basic information: Patient A, 48 years old woman, FIGO stage III, CA125 level did not return to normal after surgery.

Clinical data: The treatment plan was TC chemotherapy. Postoperative follow-up showed no obvious complications. The CA125 level did not fall within the normal range after chemotherapy.

Imaging data: MRI images showed a large tumor volume, blurred tumor margins, and some tumor tissues had invaded the surrounding tissues.

Model prediction results:

Recurrence prediction probability: 74%.

Model recommendation: Based on the recurrence risk prediction, it is recommended to intensify chemotherapy and conduct regular imaging examinations.

Discussion: In this patient’s case, the model conducted a comprehensive assessment by integrating its clinical data (such as treatment plan, CA125 level) and imaging data (such as tumor volume, invasiveness). The model predicted a high recurrence risk (74%), and based on this result, it recommended a more aggressive treatment strategy. This prediction provides strong decision support for clinicians.

#### 3.5.2 Case 2: Patient B

Basic information: Patient B, 60 years old woman, FIGO stage II, CA125 level returned to normal after surgery.

Clinical data: The treatment plan was TC chemotherapy. The postoperative complications were mild, and CA125 returned to normal levels after chemotherapy.

Imaging data: The MRI images show that the tumor volume is small, the lesion area is clear, and there is no obvious spread.

Model prediction results:

Recurrence prediction probability: 12%.

Model recommendation: Continue observation, maintain regular follow-up, and it is recommended to conduct an imaging examination every three months.

Discussion: The case of patient B indicates that although the patient has a certain medical history and the CA125 level returns to normal after treatment, the imaging data shows a stable state of the tumor. The model, by integrating the patient’s clinical data and imaging data, predicts a low recurrence probability (12%), and based on this low-risk result, recommends maintaining observation and regular follow-up.

#### 3.5.3 Case 3: Patient C

Basic information: Patient C, 55 years old woman, FIGO stage IV. Postoperative chemotherapy did not show significant improvement, and the CA125 level remained persistently high.

Clinical data: The treatment plan was TC plus bevacizumab. No obvious complications were observed after the surgery, and the CA125 level continued to rise.

Imaging data: CT imaging showed obvious angiogenesis in most areas of the tumor, suggesting a possible sign of recurrence.

Model prediction results:

Recurrence prediction probability: 90%.

Model recommendation: It is recommended to undergo further targeted treatment, strengthen follow-up and imaging monitoring, and consider whether surgical treatment is necessary.

Discussion: The case of Patient C demonstrates a situation with a high risk of recurrence. Although chemotherapy and bevacizumab treatment did not achieve the desired effect, the vascularization features on imaging suggest that the tumor may have recurred. The model combined clinical data (such as treatment plan, CA125 level) and imaging data (such as tumor angiogenesis and spread signs) to predict a recurrence probability of 90%. Based on this prediction result, the model recommends targeted treatment and strengthened follow-up.

The presentation of these actual cases demonstrates that the model can flexibly handle various clinical and imaging data and provide personalized recurrence prediction results for each patient. Through the comprehensive analysis of the patient’s clinical data, imaging data, and biomarkers, the model cannot only accurately predict the probability of recurrence but also provide scientific treatment suggestions for doctors, thereby effectively guiding clinical decisions. Through this multimodal data fusion approach, the model has shown strong adaptability and accuracy in handling complex medical data, providing reliable decision support tools for clinical doctors.

## 4 Discussion

### 4.1 Key findings and model advantages

In this exploratory proof-of-concept study, we found that a multimodal AI model integrating clinical data, medical imaging, and a knowledge graph achieved strong internal predictive performance for platinum-resistant recurrence in ovarian cancer. The proposed model, which combined a clinical MLP expert, an imaging ViT expert, and a GraphRAG-based medical knowledge expert within a Mixture of Experts (MoE) gating framework, achieved an accuracy of 0.98 (95% CI: 0.96–1.00), recall of 0.95 (95% CI: 0.92–0.98), F1 score of 0.98 (95% CI: 0.97–0.99), and AUC of 0.96 (95% CI: 0.95–0.97) in patient-level nested cross-validation on a single-center cohort of 214 patients. The model also showed good calibration (Brier score 0.042, Hosmer-Lemeshow *p* = 0.31) and positive net benefit across clinically relevant threshold probabilities in decision curve analysis.

These results, while promising from a methodological standpoint, are preliminary and require external validation before any conclusions about generalizability can be drawn. The multimodal model outperformed single-expert models and conventional benchmark models in this internal validation setting. Ablation analyses confirmed that each component contributed non-redundant value. Nevertheless, all performance estimates are derived from a single-center retrospective design and are likely optimistic. No claim of clinical readiness is made.

From a methodological perspective, the study makes several contributions. First, it demonstrates the feasibility of integrating heterogeneous data sources for a clinically relevant prediction task in ovarian cancer. Second, the MoE framework with patient-specific gating showed improved performance over static fusion. Third, the GraphRAG-based knowledge retrieval system successfully injected external biological priors without introducing detectable noise. Fourth, the comprehensive evaluation provides a rigorous benchmark for future multimodal prediction models in oncology.

However, the following methodological caveats must be emphasized. All performance metrics are derived from internal validation on a single-center dataset (*n* = 214). Internal validation is known to produce optimistic estimates compared to external test performance. Therefore, the reported AUC of 0.96 likely overestimates the model’s true generalizability. Additionally, the model’s complexity – requiring structured clinical data, high-quality imaging, and a curated knowledge graph – limits its reproducibility and scalability. These performance metrics, while encouraging, must be interpreted with caution due to the high risk of overfitting inherent in single-center retrospective studies, as discussed in detail below.

### 4.2 Limitations

Despite the rigorous methodological safeguards—including patient-level data splitting, nested cross-validation, dropout (0.5), L2 regularization, and early stopping—several important limitations must be acknowledged. The most critical limitations are the absence of external validation and the high risk of residual overfitting inherent to single-center retrospective datasets.

#### 4.2.1 Lack of external validation (most critical limitation)

This study has not been externally validated. All performance metrics are derived from patient-level nested cross-validation on a single-center cohort of 214 patients. Performance from internal validation is known to be optimistic relative to external test performance. Therefore, the reported metrics likely overestimate the model’s generalizability. We have preliminarily contacted two potential partner centers to plan future external validation, but no data have been collected or analyzed at this stage due to incomplete data sharing agreements and ethical approvals. The model is not ready for clinical deployment without independent external validation. Accordingly, this model should be considered preliminary and proof-of-concept only; it is not clinically generalizable at present.

#### 4.2.2 Single-center and small sample size

This study was conducted at a single institution (Ren Ji Hospital, Shanghai, China), which may limit the generalizability of the findings due to potential center-specific biases in patient population, treatment protocols, imaging equipment, and data collection procedures. The relatively small sample size (214 patients) further limits the precision of performance estimates and increases the risk of overfitting, despite our regularization efforts. Multi-center data with larger sample sizes are needed to assess the model’s robustness across different clinical settings.

#### 4.2.3 Gap from real clinical decisions

The model currently operates as a static risk stratification tool based on baseline and early treatment data. It does not fully capture the complexity of real-world multidisciplinary tumor board (MDT) decision-making, which integrates dynamic factors such as patient performance status, treatment tolerability, patient preferences, emerging toxicities, availability of clinical trials, and evolving biomarker results (e.g., serial ctDNA monitoring). The model also cannot simulate the trade-offs that clinicians consider (e.g., risk of adverse events versus potential benefit of aggressive therapy).

#### 4.2.4 Risk of residual overfitting and optimistic performance estimation

Despite nested cross-validation and regularization, the reported AUC of 0.96 is likely optimistic. Internal validation on a single-center retrospective cohort of 214 patients is known to produce upwardly biased estimates; average AUC drops of 0.10–0.15 are typical when moving to external validation. The model’s high parameter count (millions) further increases the risk of overfitting to institution-specific artifacts rather than generalizable signals. Therefore, the true generalizable AUC could be substantially lower (potentially 0.81–0.86), and external validation is mandatory.

### 4.3 Future direction

#### 4.3.1 External validation plan

To address the lack of external validation, we have initiated preliminary discussions with two independent institutions for future collaborative validation. Formal data sharing agreements and ethical approvals are still pending. Once these are obtained, we will conduct external validation on their cohorts (expected total sample size ~200–300 patients) and report the results separately. Until such validation is completed, the current model remains a proof-of-concept and should not be used in clinical practice.

#### 4.3.2 Integration of dynamic data with clinical workflow

To bridge the gap with real-world decision-making, future iterations of the model should: (1) Accept time-series data (e.g., CA125 kinetics during each chemotherapy cycle, imaging changes after neoadjuvant therapy) to enable dynamic risk updating. (2) Integrate patient-reported outcomes and toxicity grading (CTCAE) to reflect treatment tolerance. (3) Provide explainable outputs that can be easily interpreted by MDT members (oncologists, radiologists, pathologists).

#### 4.3.3 Prospective clinical efficacy evaluation

Real-world clinical applicability requires not only statistical generalizability but also prospective evaluation of clinical utility (e.g., impact on treatment decisions and patient outcomes). Future prospective interventional studies are needed to assess whether model-guided risk stratification improves progression-free survival or quality of life in ovarian cancer patients. These steps are essential for the model to gain clinical acceptance and regulatory approval.

### 4.4 Clinical realism and practical applicability

To realistically assess whether the proposed multimodal system could be translated into routine ovarian cancer care, we discuss four practical considerations: data availability, interpretability, computational complexity, and implementation barriers across different clinical settings.

#### 4.4.1 Availability of multimodal data in real-world practice

The model requires three data types for each patient: (1) structured and unstructured clinical data (e.g., CA125 kinetics, BRCA/HRD status, chemotherapy details), (2) high-quality CT or MRI images, and (3) a continuously updated knowledge graph. In large academic medical centers with integrated electronic health records (EHR) and PACS systems, these data are often routinely collected. However, in community hospitals or low-resource settings, critical variables (e.g., HRD status, standardized imaging protocols) may be missing. Moreover, the knowledge graph requires ongoing curation by experts, which is not available in most clinical environments. Therefore, at present, the model is only feasible in well-resourced tertiary centers that have dedicated AI support teams.

#### 4.4.2 Interpretability of model outputs

The model’s output is a risk probability (0–1) and a binary classification. To be clinically useful, clinicians need to understand why the model made a particular prediction. The MoE gating network provides some interpretability by revealing which expert (clinical, imaging, or knowledge) contributed most to each prediction. Additionally, the GraphRAG module can output retrieved knowledge subgraphs with explanatory paths (e.g., “BRCA wild-type → HRD negative → reduced PARP inhibitor sensitivity”). However, these explanations are not yet fully automated or integrated into a user-friendly interface. Without transparent, case-level reasoning, clinician trust and adoption will remain low. Future studies should focus on developing interpretable dashboards that highlight the key drivers of each risk prediction.

#### 4.4.3 Computational complexity

The model comprises several computationally intensive components: BERT (110 M parameters), ViT (86 M parameters), GraphRAG, and MoE. Inference on a single patient (including text and image processing) takes approximately 10–15 s on a modern GPU (e.g., NVIDIA A100). This may be acceptable for batch processing or research, but is challenging for real-time clinical decision support. Cloud-based deployment could reduce local hardware requirements, but then data privacy (e.g., patient confidentiality, HIPAA/GDPR compliance) becomes a major concern. On-premise GPU clusters are expensive and require specialized IT support, limiting accessibility for smaller institutions.

#### 4.4.4 Implementation barriers across clinical settings

Several barriers must be overcome before routine use:

Integration with EHR/PACS: Automated data extraction pipelines are needed; manual data entry is infeasible.
Regulatory approval: The model would require regulatory clearance (e.g., NMPA, FDA) after successful external validation – a multi-year process.
Workflow integration: The model’s output must be presented at the right time (e.g., before starting second-line therapy) and in a format that fits existing multidisciplinary tumor board workflows.
Acceptance by clinicians: Many clinicians are skeptical of “black-box” AI models. Pilot studies with prospective but non-interventional use are needed to build trust.
Cost: GPU hardware, software licenses, and AI engineering time are expensive. Without demonstrated cost-effectiveness, adoption will be limited.

#### 4.4.5 Summary on feasibility

In its current form, the model is not feasible for routine clinical practice. It is a research prototype that requires substantial resources, technical expertise, and institutional support. Future efforts should focus on simplifying the model (e.g., reducing to the top-10 clinical features and a single imaging sequence), automating data extraction, improving interpretability, and conducting prospective feasibility pilots. Only after successful external validation and workflow integration could clinical implementation be considered.

## 5 Conclusion

In summary, this exploratory proof-of-concept study demonstrates that a multimodal AI model integrating clinical, imaging, and knowledge graph data can achieve strong internal predictive performance for platinum-resistant recurrence in ovarian cancer in a single-center retrospective cohort. However, the study has major limitations: no external validation, small sample size (*n* = 214), single-center retrospective design, and substantial data and computational requirements. The model should be considered a hypothesis-generating tool and a methodological demonstration of multimodal fusion. It is not ready for clinical practice. Independent multi-center validation is mandatory before any clinical translation can be considered.

## Data Availability

The original contributions presented in the study are included in the article/supplementary material, further inquiries can be directed to the corresponding authors.

## References

[1] PDQ Cancer Genetics Editorial Board. Cancer Genetics Risk Assessment and Counseling (PDQ®)–Health Professional Version. Bethesda, MD: National Cancer Institute. (2002) 26389258

[2] Smolarz B Biernacka K Łukasiewicz H Samulak D Piekarska E Romanowicz H . Ovarian cancer-epidemiology, classification, pathogenesis, treatment, and estrogen receptors’ molecular backgrounds. Int J Mol Sci. (2025) 26:4611. doi: 10.3390/ijms26104611, 40429755 PMC12111435

[3] La Vecchia C. Ovarian cancer: epidemiology and risk factors. Eur J Cancer Prev. (2017) 26:55–62. doi: 10.1097/CEJ.0000000000000217. 26731563, 26731563

[4] El Bairi K Singh S Le Page C. Revisiting platinum-resistant ovarian cancer: advances in therapy, molecular biomarkers, and clinical outcomes. Semin Cancer Biol. (2021) 77:1–2. doi: 10.1016/j.semcancer.2021.09.002, 34492299

[5] St Laurent J Liu JF. Treatment approaches for platinum-resistant ovarian Cancer. J Clin Oncol. (2024) 42:127–33. doi: 10.1200/JCO.23.01771. 37910841, 37910841

[6] Havasi A Cainap SS Havasi AT Cainap C. Ovarian cancer-insights into platinum resistance and overcoming it. Medicina (Kaunas). (2023) 59:544. doi: 10.3390/medicina59030544, 36984544 PMC10057458

[7] El Bairi K Madariaga A Trapani D Al Jarroudi O Afqir S. New horizons for platinum-resistant ovarian cancer: insights from the 2023 American Society of Clinical Oncology (ASCO) and European Society for Medical Oncology (ESMO) annual meetings. Int J Gynecol Cancer. (2024) 34:760–72. doi: 10.1136/ijgc-2023-004927. 38101815, 38101815

[8] Nougaret S McCague C Tibermacine H Vargas HA Rizzo S Sala E . Radiogenomics in ovarian cancer: a literature review. Abdom Radiol (NY). (2021) 46:2308–2322. doi: 10.1007/s00261-020-02820-z 33174120

[9] Yu KH Healey E Leong TY Kohane IS Manrai AK. Medical artificial intelligence and human values. N Engl J Med. (2024) 390:1895–904. doi: 10.1056/NEJMra2214183, 38810186 PMC12425466

[10] Lenharo M. An AI revolution is brewing in medicine. What will it look like? Nature. (2023) 622:686–8. doi: 10.1038/d41586-023-03302-0

[11] Singh R Bapna M Diab AR Ruiz ES Lotter W. How AI is used in FDA-authorized medical devices: a taxonomy across 1,016 authorizations. NPJ Digit Med. (2025) 8:388. doi: 10.1038/s41746-025-01800-1, 40596700 PMC12219150

[12] Piliuk K Tomforde S. Artificial intelligence in emergency medicine. A systematic literature review. Int J Med Inform. (2023):180. doi: 10.1016/j.ijmedinf.2023.105274 37944275

[13] Liu Z Wu J Gao X Qin Z Tian R Wang C. Deep learning-based automatic measurement system for patellar height: a multicenter retrospective study. J Orthop Surg Res. (2024) 19:324. doi: 10.1186/s13018-024-04809-6, 38822361 PMC11141039

[14] Bai J-W Qiu S-Q Zhang G-J. Molecular and functional imaging in cancer-targeted therapy: current applications and future directions. Signal Transduct Target Ther. (2023) 8:89. doi: 10.1038/s41392-023-01366-y 36849435 PMC9971190

[15] Qi L Wang W Wu T Zhu L He L Wang X. Multi-omics data fusion for Cancer molecular subtyping using sparse canonical correlation analysis. Front Genet. (2021) 12:607817. doi: 10.3389/fgene.2021.607817, 34367231 PMC8341864

[16] Gao Y Zeng S Xu X Li H Yao S Song K . Deep learning-enabled pelvic ultrasound images for accurate diagnosis of ovarian cancer in China: a retrospective, multicentre, diagnostic study. Lancet Digit Health. (2022) 4:e179–87. doi: 10.1016/S2589-7500(21)00278-8, 35216752

[17] Azizi S Culp L Freyberg J Mustafa B Baur S Kornblith S . Robust and data-efficient generalization of self-supervised machine learning for diagnostic imaging. Nat Biomed Eng. (2023) 7:756–79. doi: 10.1038/s41551-023-01049-7, 37291435

[18] Yang H Yang M Chen J Yao G Zou Q Jia L. Multimodal deep learning approaches for precision oncology: a comprehensive review. Brief Bioinform. (2024) 26:bbae699. doi: 10.1093/bib/bbae699, 39757116 PMC11700660

[19] Li X Ma J Leng L Han M Li M He F . MoGCN: a multi-omics integration method based on graph convolutional network for Cancer subtype analysis. Front Genet. (2022) 13:806842. doi: 10.3389/fgene.2022.806842, 35186034 PMC8847688

[20] Wu Y Wang S Song G Huang Q. Augmented adversarial training for cross-modal retrieval. IEEE Trans Multimed. (2020) 23:559–71. doi: 10.1109/TMM.2020.2985540

[21] Brown TB Mann B Ryder N Subbiah M Kaplan J Dhariwal P . MedVH: toward systematic evaluation of hallucination for large vision language models in the medical context. Adv Intell Syst. (2025) 8:1–22. doi: 10.1002/aisy.202500255, 40843006 PMC12363988

